# Nitrogen Sources Affect the Long-Chain Polyunsaturated Fatty Acids Content in *Thraustochytrium* sp. RT2316-16

**DOI:** 10.3390/md21010015

**Published:** 2022-12-25

**Authors:** Diego Valdebenito, Sebastián Urrutia, Allison Leyton, Yusuf Chisti, Juan A. Asenjo, Carolina Shene

**Affiliations:** 1 Center for Biotechnology and Bioengineering (CeBiB), Center of Food Biotechnology and Bioseparations, BIOREN and Department of Chemical Engineering, Universidad de La Frontera, Francisco Salazar 01145, Temuco 4780000, Chile; 2 Institute of Tropical Aquaculture and Fisheries, Universiti Malaysia Terengganu, Kuala Nerus 21030, Terengganu, Malaysia; 3 Centre for Biotechnology and Bioengineering (CeBiB), Department of Chemical Engineering, Biotechnology and Materials, Universidad de Chile, Beauchef 851, Santiago 8370459, Chile

**Keywords:** *Thraustochytrium* sp., carotenoids, omega-3 polyunsaturated fatty acids, microbial lipids, amino acids

## Abstract

The psychrophilic marine microorganism *Thraustochytrium* sp. RT2316-16 can produce carotenoids as well as lipids containing the omega-3 polyunsaturated fatty acids (PUFA) eicosapentaenoic acid and docosahexaenoic acid. This work reports on the effects of the composition of the culture medium, including certain amino acids, on growth and lipid synthesis by RT2316-16. Compared with the culture on glutamate, the use of lysine, alanine, or serine, increased the content of the omega-3 PUFA in total lipids. In the media that contained yeast extract, glutamate, and glucose, lipid accumulation occurred when organic ammonium was exhausted earlier than glucose. In contrast, lipid mobilization was promoted if glucose was exhausted while organic ammonium (supplied by yeast extract and glutamate) remained in the medium. The total content of carotenoids in the lipid-free biomass decreased during the first 12 to 24 h of culture, simultaneously with a decrease in the total lipid content of the biomass. The experimental data suggested a possible interrelationship between the metabolism of carotenoids and lipids. A high content of omega-3 PUFA in the total lipids could be obtained by growing the thraustochytrid in a medium with a low glucose concentration (6 g L^−1^) and a high concentration of organic nitrogen (yeast extract 12 g L^−1^; glutamate 1.06 g L^−1^), after glucose was exhausted. These observations may guide the development of a strategy to enhance omega-3 PUFA in the biomass.

## 1. Introduction

The long-chain omega-3 polyunsaturated fatty acids (PUFA), eicosapentaenoic acid (C20:5n-3; EPA), docosapentaenoic acid (C22:5n-3; DPA), and docosahexaenoic acid (C22:6n-3; DHA), are metabolically important in humans. For example, DHA is required for brain development and functioning. Additionally, DHA occurs in diverse cell membranes and affects cellular metabolism [1,2]. EPA, DPA, and DHA are made in the human body but at low rates, and their dietary intake provides various health benefits [3]. Oils rich in DHA and EPA are widely used in human nutrition.

A number of marine thraustochytrids accumulate high levels of lipids (>40% of cell dry weight) rich in EPA and DHA. Among thraustochytrids, strains of the genus *Aurantiochytrium* are particularly attractive as they produce DHA via polyketide synthase (PUFA–PKS), avoiding the synthesis of intermediates in the PUFA pathway [4]. In some strains of *Thraustochytrium*, DHA synthesis occurs through a combination of elongase–desaturase enzymes and the PUFA–PKS system [5]. Lipids in these strains have a more complex fatty acid profile.

*Thraustochytrium* sp. RT2316-16 is a psychrophilic marine protist that does not grow at temperatures exceeding 15 °C. RT2316-16 produces carotenoids (β-carotene, canthaxanthin and astaxanthin) and lipids that contain EPA and DHA [6]. Carotenoids, such as β-carotene (a precursor of vitamin A in humans), are essential nutrients for humans. The DHA and EPA content in RT2316-16 depends strongly on the mode of culture (batch or repetitive batch) and the composition of the culture medium. Media for growing RT2316-16 typically require a carbon source (glucose or glycerol), nitrogen sources and vitamins. The vitamins that are essential have not been established. Yeast extract and glutamate are the most commonly used nitrogen sources for evaluating thraustochytrids for the production of DHA, as these nitrogen sources support good biomass growth [7]. The yeast extracts contain mainly amino acids and peptides that may be used directly by microorganisms for protein synthesis. Although thraustochytrids may be able to live on inorganic nitrogen sources such as ammonium and nitrate [8], the presence of amino acids in the medium supports rapid proliferation.

This work reports on the contributions of glucose, yeast extract, and glutamate to the production of biomass and lipids by RT2316-16, as these data are necessary for defining feeding strategies in fed-batch cultures intended to maximize the productivity of DHA and carotenoids. Although the results confirmed the ability of RT2316-16 to grow using only glutamate and glucose, the potential of the other 19 amino acids for promoting growth and lipid production was investigated. The amino acid metabolism of thraustochytrids has not been previously discussed. The production of total lipids (TL) and total carotenoids (TC) in media differing in initial concentrations of glucose and yeast extract-glutamate is discussed.

The results revealed that the design of the culture medium had a profound impact on the omega-3 PUFA content of the biomass of RT2316-16. The media used here may be useful in enhancing the production of total lipids and PUFA in other similar thraustochytrids. In addition, the observed changes in the fatty acid composition of the total lipids during incubation can be used to decide on the optimal harvest time to maximize the fraction of omega-3 PUFA.

## 2. Results

### 2.1. Effect of the Organic Nitrogen Source on Production of Biomass and Lipids by RT2316-16

**Figure 1:**
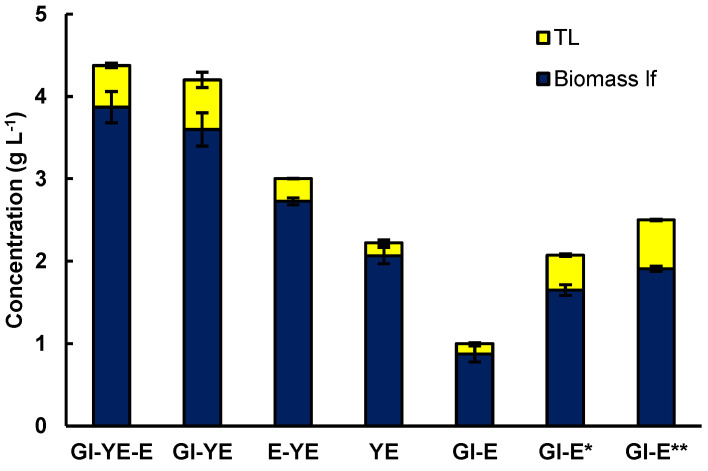
Effect of the substrates (glucose (Gl) 20 g L^−1^, yeast extract (YE) 6 g L^−1^ and glutamate (E) 0.52 g L^−1^) in the M medium used for the cultivation of RT2316-16 on the concentrations of lipid-free biomass (Biomass lf) and total lipids (TL). Gl-E* and Gl-E** denote the results for a medium that contained only glucose (20 g L^−1^) and glutamate at concentrations of 2.1 and 3.1 g L^−1^, respectively. Incubation conditions were 5 days at 15 °C and 150 rpm on the shaker platform.

As a first objective, the effects of the different media components (glucose, yeast extract, and glutamate) on the production of biomass and lipids by RT2316-16 were elucidated. The basal medium M is comprised of glucose (20 g L^−1^), yeast extract (6 g L^−1^), and monosodium glutamate (0.6 g L^−1^) (0.52 g L^−1^ glutamic acid) [6,9,10]. As shown in Figure 1, eliminating glutamate from this medium had no significant effect (*p* > 0.05) on the biomass concentration (4.4 ± 0.1 g L^−1^ and 4.2 ± 0.6 g L^−1^ with and without glutamate, respectively). Glutamate had a small but significant effect (*p* < 0.05) on the lipid content of the biomass (11.6 ± 1.2% and 14.3 ± 0.8 % w w^−1^, with and without glutamate, respectively).

RT2316-16 used yeast extract as the only source of carbon and nitrogen for producing a biomass (2.1 ± 0.1 g of lipid-free biomass L^−1^) with a low lipid content (7.1 ± 0.6% w w^−1^). Supplementing the yeast extract with glutamate increased the concentration of the lipid-free biomass (32% increase) and the total lipids in the biomass (29% increase) (Figure 1). Additionally, the lowest concentration of lipid-free biomass (0.9 ± 0.1 g L^−1^) was obtained in a medium that contained both glucose and glutamate. This would be due to growth limited by nitrogen because when glutamate concentration was 2.1 g L^−1^ the concentration of the lipid-free biomass and the total lipids in the biomass attained values of 1.6 ± 0.1 g L^−1^ and 20.5% w w^−1^, respectively. The concentration of the lipid-free biomass and the total lipids in the biomass were 1.9 ± 0.1 g L^−1^ and 23.6% w w^−1^, respectively, when the glutamate concentration was 3.1 g L^−1^.

**Figure 2:**
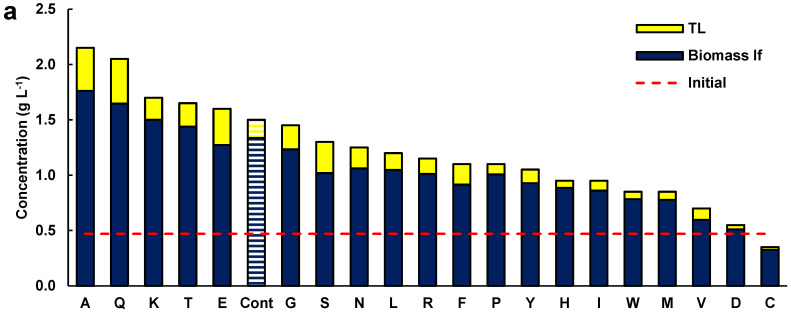

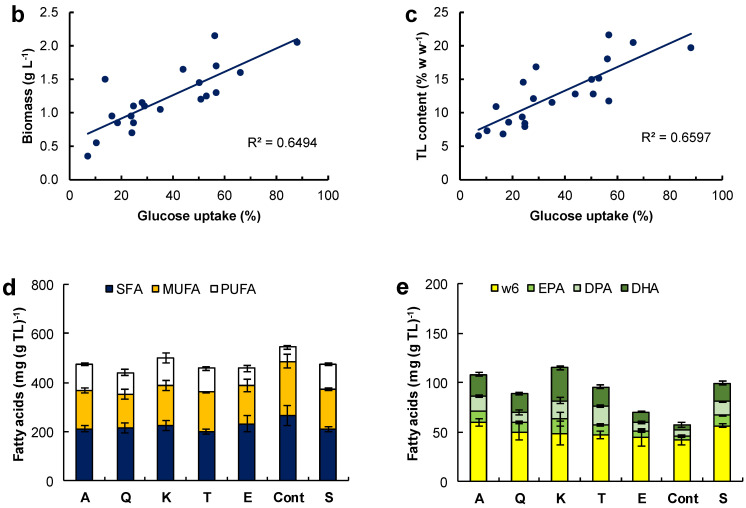
(**a**) Concentration of lipid-free biomass (Biomass lf) and total lipids (TL) in media containing glucose (5 g L^−1^), 1 g L^−1^ of the respective amino acid (alanine A, glutamine Q, lysine K, threonine T, monosodium glutamate E, glycine G, serine S, asparagine N, leucine L, arginine R, phenylalanine F, proline P, tyrosine Y, histidine H, isoleucine I, tryptophan W, methionine M, valine V, aspartic acid D, and cysteine C), and 6.7 g L^−1^ YBN. Cont is the control medium without amino acids. (**b**) Relationship between glucose uptake and total biomass concentration. (**c**) Relationship between glucose uptake and total lipid content of the biomass. Composition of (**d**) the fatty acids and (**e**) polyunsaturated fatty acids in the TL from the biomass grown with a single amino acid. Incubation conditions were 2 days at 15 °C and 150 rpm.

As the effect of glutamate in the presence of glucose was significant on the growth and lipid content of the biomass, the effects of the 20 amino acids (each at 1 g L^−1^) were tested individually in media formulated with glucose (5 g L^−1^) and Yeast Nitrogen Base (YNB) Without Amino Acids (Difco, BD 291940) (Figure 2a). The YNB provided vitamins and salts, including ammonium sulfate (5 g L^−1^). In this set of experiments, a control medium was included which contained glucose and YNB.

The concentration of the lipid-free biomass and the total lipids in the biomass of these experiments (Figure 2) cannot be compared with the data shown in Figure 1, because of the different concentrations of glucose and the shorter incubation time (48 h). The biomass used to inoculate cultures for obtaining the data in Figure 2 was grown in the medium M. To prevent the residual medium from influencing the results, the biomass was recovered by centrifugation and washed twice with sterile, diluted artificial seawater. Additionally, all the experiments were inoculated with the same culture, and differences in the initial biomass concentration in the experiments were not significant (*p* > 0.05; not shown).

Five amino acids (alanine, glutamine, lysine, threonine, and glutamate) resulted in a higher concentration of lipid-free biomass relative to the control (1.3 g L^−1^) (Figure 2a). In addition, these amino acids resulted in higher total lipid concentrations in the cultures relative to the control experiment (0.16 g L^−1^). The media with glycine, serine, asparagine, and phenylalanine did not raise the concentration of biomass but allowed a higher concentration of total lipids in comparison with the control (Figure 2a). Therefore, beside glutamate (Figure 1), several other amino acids had a beneficial role in biomass and lipid production by RT2316-16.

In media with the different amino acids, the consumption of glucose varied between 7.1 and 88%, depending on the amino acid. A positive correlation between biomass concentration (Figure 2b; R^2^ = 0.649) and glucose consumption and between total lipids in the biomass and glucose consumption (Figure 2c; R^2^ = 0.660) was determined. The data used in these graphs is shown in Table S1 (Supplemental Material). The consumption of the amino acids was not measured because one of the vitamins in YNB interfered with the method used for amino acid analysis [11].

The fatty acid composition of the total lipids extracted from the biomass grown with alanine, glutamine, lysine, threonine, glutamate, serine, and the control medium (without amino acids) is shown in Figure 2d (contents of saturated fatty acids, SFA; monounsaturated fatty acids, MUFA; and PUFA), and Figure 2e (contents of EPA, DPA, and DHA; and omega-6 PUFA). The full fatty acid profiles of the total lipids are shown in Table S2 (Supplemental Material). The total lipids with the highest content of fatty acids were those from the biomass grown in the control medium (545 ± 78 mg (g TL)^−1^), but a statistically identical (*p* > 0.05) result (502 ± 62 mg (g TL)^−1^) was found for the lipids extracted from the biomass grown with lysine (Figure 2d). The lowest (57 ± 9 mg (g TL)^−1^) and the highest (115 ± 21 mg (g TL)^−1^) content of PUFA were found in the total lipids from the biomass grown in the control medium and in the medium with lysine, respectively (Figure 2e). The main PUFA in the total lipids extracted from the biomass grown with alanine, glutamine, lysine, threonine, glutamate, serine, and the control were the omega-6 PUFA (linoleic acid C18:2n-6, γ-linolenic acid C18:3n-6, and dihomo-γ-linolenic acid C20:3n-6). EPA, DPA, and DHA were also found in the PUFA fraction. The biomass grown in the control medium (no amino acids) had the lowest content of all the omega-3 PUFA in its lipids (14.3 ± 4.3 mg (g TL)^−1^) (Figure 2e).

### 2.2. Effect of the Initial Concentrations of Glucose and Organic Nitrogen Source

**Figure 3:**
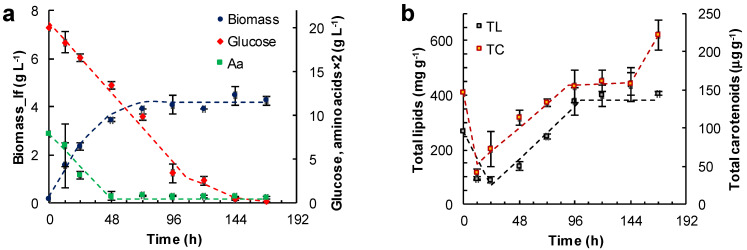

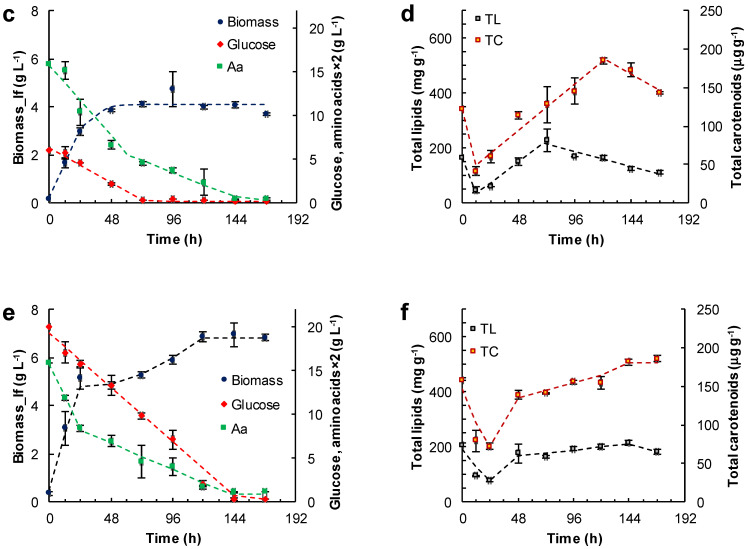
Effect of the medium composition and incubation time on the concentration of the lipid-free biomass (Biomass) (**a**,**c**,**e**) and the content of total lipids (TL) and carotenoids (TC) (**b**,**d**,**f**) in RT2316-16 biomass. Concentrations of glucose and amino acids (Aa) (from yeast extract and glutamate) are also shown (**a**,**c**,**e**). Initial concentration of glucose, yeast extract and monosodium glutamate: 20, 6 and 0.6 g L^−1^ (medium M, (**a**,**b**)); 6, 12 and 1.2 g L^−1^ (medium ML, (**c**,**d**)); 20, 12 and 1.2 g L^−1^ (medium MH, (**e**,**f**)), respectively. Incubation conditions were: 15 °C and 150 rpm.

The aim of this set of experiments was to establish if the initial concentration of glucose and the organic nitrogen source (yeast extract–glutamate) had any effects on the concentration of lipid-free biomass and the total lipids and carotenoids in lipid-free biomass (Figure 3).

The lipid-free biomass (lfb) was used as the uniform basis for comparing the contents of lipids and carotenoids. The growth media evaluated were: the medium M (Figure 3a,b), the medium ML (glucose 6 g L^−1^, yeast extract 12 g L^−1^, glutamate 1.04 g L^−1^) (Figure 3c,d) and the medium MH (glucose 20 g L^−1^, yeast extract 12 g L^−1^, glutamate 1.04 g L^−1^) (Figure 3e,f). The amino acid composition of the yeast extract used in the media is shown in Table S3 (Supplemental Material). Thus, glutamate concentration in the media M, ML and MH was 0.86, 1.71 and 1.71 g L^−1^, respectively. In these experiments the concentration of lipid-free biomass increased almost linearly in the first few hours of incubation, indicating an absence of an adaptation period, or the lag phase of growth. The initial growth rates (r_Xlf_, g L^−1^ h^−1^) of lipid-free biomass in various media are shown in Table 1.

The absence of a lag phase in the growth curves (Figure 3a,c,e) was because the inoculum had been prepared using the same medium as that used to obtain the growth curves. Thus, the microorganism did not require any adaptation. During the period of linear growth, the concentration of glucose and amino acids (from yeast extract and glutamate) also decreased almost linearly with time. The consumption rates of glucose (−r_S_) and amino acids (−r_AA_) are shown in Table 1.

In experiments with the medium M, the concentration of the lipid-free biomass ceased to increase once amino acids were exhausted (48 h) (Figure 3a), and the maximum concentration attained was 4.2 ± 0.4 g L^−1^ (Figure 3a). The glucose concentration continued to decrease until 144 h, and the rate of consumption was 0.17 g (L h)^−1^ between 0 and 96 h. The total lipids in lipid-free biomass increased from 68 mg (g lfb)^−1^ at 24 h to 380 mg (g lfb)^−1^ at 144 h (Figure 3b), while glucose was available (Figure 3a). In addition, the total carotenoids in the lipid-free biomass increased stepwise from 69 μg (g lfb)^−1^ at 24 h to 160 μg (g lfb)^−1^ at 144 h (Figure 3b).

In experiments with the ML medium, the lipid-free biomass concentration increased between 0 and 48 h of incubation to attain a peak value of 4.1 ± 0.1 g L^−1^ (Figure 3c). Subsequently, the glucose was exhausted after 72 h of incubation. At this time the uptake rate of amino acids decreased from 0.84 × 10^−1^ to 0.29 × 10^−1^ g (L h)^−1^ (Figure 3c, Table 1). Glucose exhaustion determined the transition from a phase in which the total lipid content increased to a phase in which the total lipid decreased (Figure 3d). The total carotenoids in the lipid-free biomass increased from 12 h (50 μg (g lfb)^−1^) to 121 h (187 μg (g lfb)^−1^) (Figure 3d), but declined once the concentration of amino acids decreased to 0.2 g L^−1^ (Figure 3c).

The highest concentration of the lipid-free biomass (6.8 ± 0.1 g L^−1^) was reached in the MH medium after 168 h of incubation (Figure 3e). There were two growth phases observed in this experiment: in the first phase of linear growth (0–24 h), the concentration of the lipid-free biomass increased 14.3-fold at a rate of 0.2 g (L h)^−1^ (Table 1); in the second phase (24–120 h), slower growth occurred until amino acids were exhausted (Figure 3e). The uptake rate of amino acids was different in the two growth phases (Figure 3e; Table 1).

The rates of accumulation of total lipids and total carotenoids in the lipid-free biomass were different during the different phases of growth (Figure 3f; Table 1). The uptake rate of amino acids had barely any effect on the rate of consumption of glucose (0.13 g (L h)^−1^). The total carotenoids in the final lipid-free biomass in MH medium were not significantly different (*p* > 0.05) compared to the maximum carotenoids in the lipid-free biomass grown in ML medium (Figure 3d). The MH medium produced biomass with the lowest total lipids (<200 mg (g lfb)^−1^) (Figure 3e). The total lipid content decreased once glucose was exhausted (after 144 h) (Figure 3f).

### 2.3. Effect of Other Carbon Sources

**Figure 4:**
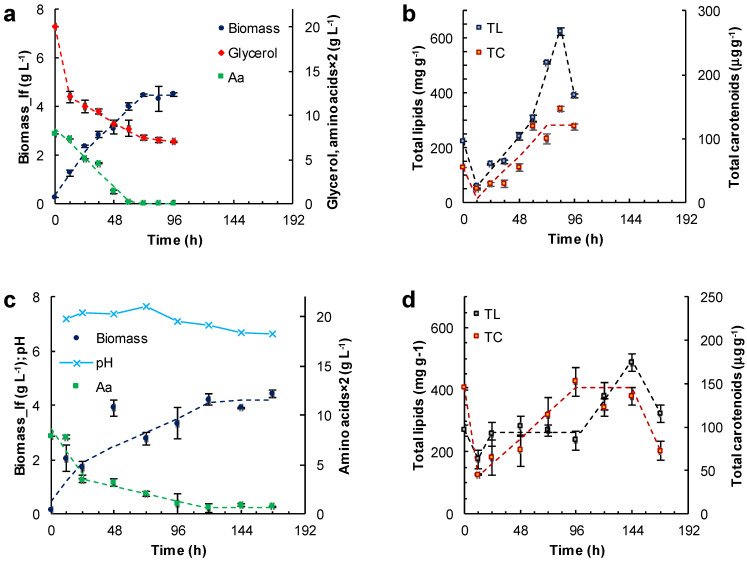
Effect of the other carbon sources and incubation time on the concentration of the lipid-free biomass (Biomass) (**a**,**c**) and the content of total lipids (TL) and carotenoids (TC) (**b**,**d**) in RT2316-16 biomass. Concentration of amino acids (Aa) (from yeast extract and glutamate) is also shown (**a**,**c**). Initial concentrations were: (**a**,**b**) glycerol (20 g L^−1^); (**c**,**d**) canola oil (20 g L^−1^). All cultures (**a**–**d**) contained yeast extract (6 g L^−1^) and monosodium glutamate (0.6 g L^−1^). Incubation conditions were: 15 °C and 150 rpm.

Some experiments were carried out with the glucose in the M medium replaced with either glycerol (Figure 4a,b) or canola oil (Figure 4c,d).

In the medium formulated with glycerol, two growth phases of the lipid-free biomass were observed (0–24 h; 24–72 h) (Figure 4a; Table 1). The final concentration of the lipid-free biomass was 4.5 ± 0.2 g L^−1^. The rate of glycerol consumption decreased from 0.08 to 0.02 g (L h)^−1^ once amino acids were exhausted at 60 h (Figure 4a). The amino acid exhaustion coincided with the start of a phase (60–84 h) in which the total lipids in the lipid-free biomass increased by nearly two-fold (from 320 to 620 mg (g lfb)^−1^) in 24 h (Figure 4b; Table 1). The total carotenoids in the lipid-free biomass increased from 30 μg (g lfb)^−1^ at 12 h to 121 μg (g lfb)^−1^) at 60 h (Figure 4b).

Canola oil was used as a carbon source to test if RT2316-16 was able to metabolize triacylglycerols. In using canola oil as the carbon source, the final concentration of lipid-free biomass was 4.2 ± 0.1 g L^−1^ (Figure 4c); this was not significantly different (*p* > 0.05) from the concentration obtained with glucose (Figure 3a), or glycerol with the other components as in medium M (Figure 4a). The lipid-free biomass exhibited two growth phases (0–24 h; 24–120 h) with differing rates of amino acid consumption (Figure 4c; Table 1). Exhaustion of amino acids led to a stationary phase in lipid-free biomass concentration (Figure 4c). In this experiment, the concentration of the residual oil was not measured because it was not possible to obtain a homogeneous sample. The total lipids in lipid-free biomass remained nearly constant (260 mg (g lfb)^−1^) between 24 and 96 h (Figure 4d), but increased 1.9-fold after amino acids were exhausted (Figure 4c). During the last 24 h the total lipid content decreased from 489 ± 28 to 322 ± 29 mg (g lfb)^−1^. The total carotenoids increased 2.5-fold between 24 and 96 h but decreased to 72 μg (g lfb)^−1^ in the last 24 h (Figure 4d). The pH remained nearly constant (7.3) until 96 h (Figure 4c). Afterwards, the pH decreased with a simultaneous increase in total lipids (Figure 4d). The pH decline was likely related to the release of free fatty acids as a consequence of the hydrolysis of the triacylglycerol in the oil. Changes in pH were not seen (not shown) in experiments with glucose (Figure 3) and glycerol (Figure 4a) as carbon sources.

### 2.4. Composition of the Total Lipids in RT2316-16 during the First 48 h of Incubation

**Figure 5:**
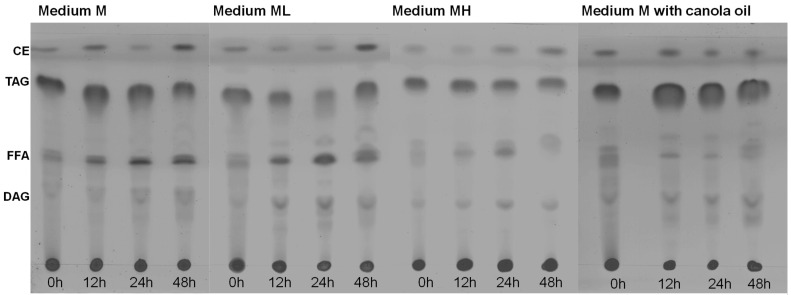
Composition of the total lipids of *Thraustochytrium* sp. RT2316-16 as a function of the incubation time (0–48 h; shown at the bottom of each chromatogram) in different media. Initial concentrations of glucose, yeast extract and glutamate 20, 6 and 0.52 g L^−1^ (the medium M); 6, 12 and 1.04 g L^−1^ (the medium ML); 20, 12 and 1.04 g L^−1^ (MH), respectively. In the canola oil experiment, the initial concentrations of canola oil, yeast extract, and glutamate were 20, 6, and 0.52 g L^−1^. CE, cholesterol esters; TAG, triacylglycerols; FFA, free-fatty acids; and DAG, diacylglycerols.

As shown in Figure 3 and Figure 4, the total lipids in the biomass decreased during the first 12 h, or 24 h, of incubation. The lipid types present in the total lipid fraction were qualitatively determined by thin layer chromatography (Figure 5) in attempts to explain the observed decline in total lipids.

At inoculation (t = 0 h), the total lipids in the biomass grown using medium M (Figure 3a,b) mainly comprised triacylglycerols and lower fractions of cholestrol esters and free-fatty acids (Figure 5). After 12 h of incubation, the free-fatty acid fraction increased. This increase in the free-fatty acid fraction and the observed decrease of the total lipid content (Figure 3b) suggested that the accumulated triacylglycerols were hydrolyzed to support metabolism. A similar behavior was observed in the composition of total lipids in the biomass grown in the ML medium. The fraction of total lipids in the biomass grown in the MH medium was lower that than in the biomass grown in the M medium and in the ML medium. This difference coincided with a lower total lipid content in the biomass grown in MH medium (Figure 3f). For the biomass grown in canola oil, the high initial content of free fatty acids was explained by the microorganism hydrolyzing the oil for use as a carbon source.

### 2.5. Composition of the Fatty Acids in the Lipids of the Biomass Grown in Different Media

**Figure 6:**
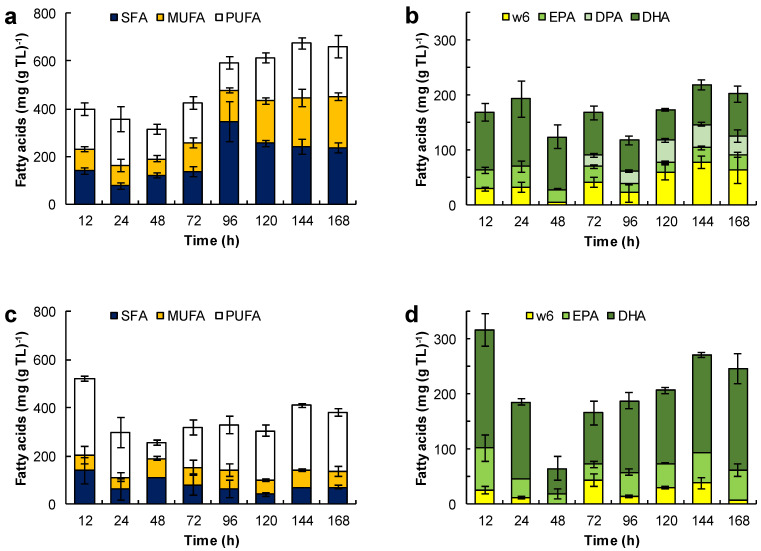

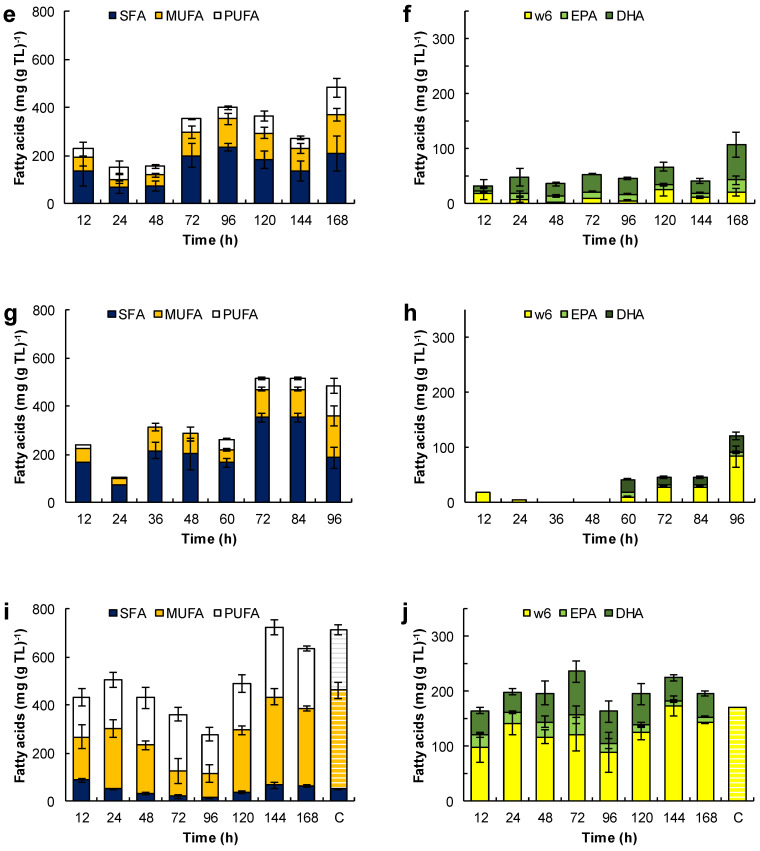
Effect of the medium composition and incubation time on the content of saturated fatty acids (SFA), monounsaturated fatty acids (MUFA) and polyunsaturated fatty acids (PUFA) (**a**,**c**,**e**,**g**,**i**) and the content of eicosapentaenoic acid (EPA), docosapentaenoic acid (DPA), docosahexaenoic acid (DHA), and omega 6 PUFA (w6) (C18:2n-6, C18:3n-6, C20:3n-6 and C20:4n-6) (**b**,**d**,**f**,**h**,**j**) in the total lipids (TL) of RT2316-16. Initial concentration of glucose, yeast extract, and monosodium glutamate: 20, 6, and 0.6 g L^−1^ (medium M, a,b); 6, 12, and 1.2 g L^−1^ (medium ML, c,d); 20, 12, and 1.2 g L^−1^ (medium MH, e,f), respectively. Experiments used medium M with the glucose replaced by glycerol (g,h) and canola oil (i,j). The striped bars (marked by C in graphs i and j) corresponded to the fatty acid composition of canola oil.

The composition of the fatty acids in the total lipids extracted from the biomass grown in the different media is shown in Figure 6. The relevant data are included in the Supplemental Material (Tables S4–S8 in the Supplemental Material).

The fatty acid composition of total lipids depended on the growth medium used and the incubation time (Figure 6). For the biomass grown in medium M, the fatty acids in the total lipid decreased from 400 to 313 mg (g TL)^−1^ between 12 h and 48 h of incubation (Figure 6 a). This loss coincided with the rapid growth of the lipid-free biomass (Figure 3a) and the initial loss in the total lipid content of the biomass (Figure 3b). Between 12 h and 24 h, the proportion of saturated fatty acids decreased, but it nearly doubled (from 122 to 345 mg (g TL)^−1^) between 48 h and 96 h of incubation. Between 96 h and 144 h, the fraction of PUFA increased 1.9-fold (from 118 to 228 mg (g TL)^−1^; Figure 6a). This increase correlated with an increase in the omega-3 PUFA, that is EPA, DPA and DHA (Figure 6b).

The total lipids of the biomass grown in the ML medium (Figure 6c) had comparatively fewer fatty acids (an average of 352 mg (g TL)^−1^) than the total lipids of the biomass grown in the M medium (Figure 6a). This was related to a lower concentration of glucose and a higher concentration of amino acids in the medium ML. These total lipids produced in the ML medium had a low proportion of SFA (an average of 79 mg (g TL)^−1^) and MUFA (an average of 67 mg (g TL)^−1^). The significant lowering of the total lipids after 72 h of incubation due to the exhaustion of glucose (Figure 3d) did not affect their fatty acid content. The fraction of DHA in the total lipids increased from 93 ± 21 mg (g TL)^−1^ at 72 h to 184 ± 28 mg (g TL)^−1^ at 168 h (Figure 6d). The omega-6 the PUFA fraction (mainly arachidonic acid and linoleic acid) was quite low (< 38 mg (g TL)^−1^).

The increase in concentration of the lipid-free biomass in the MH medium between 12 h and 24 h (Figure 3f) may have been related to the decrease of the free-fatty acid content in the total lipids (from 228 to 150 mg (g TL)^−1^), especially the decrease in the SFA fraction (from 135 to 68 mg (g TL)^−1^) (Figure 6e). Between 48 h and 96 h of incubation (that is, the second growth phase; Figure 3f), all the various fractions of fatty acids increased (Figure 6e), especially the SFA fraction. The PUFA fraction showed a moderate increase with incubation time (34 mg (g TL)^−1^ at 12 h; 110 mg (g TL)^−1^ at 168 h; Figure 6f), but its contribution was lower than in the biomass grown in the media M and ML. The main PUFA in this fraction of total lipids was DHA (10–65 mg (g TL)^−1^; Figure 6f). On the other hand, the proportion of omega-6 PUFA was low and comparable to the values observed in the lipids produced in the ML medium.

SFA were the main fatty acids in the biomass grown with glycerol (Figure 6g). During the growth, the fatty acids in lipid-free biomass remained under 300 mg (g TL)^−1^, but increased to 622 ± 13 mg (g TL)^−1^ during the stationary phase (Figure 4a). The PUFA content was low (0–47 mg (g TL)^−1^) during growth but rose to 127 mg (g TL)^−1^ in the final 24 h of culture (Figure 6h).

The Figure 6i,j show the evolution of the different fractions of fatty acids in the total lipids of the biomass grown with canola oil (Figure 4c). These lipids had a high proportion of oleic acid (100–355 mg (g TL)^−1^) and omega-6 PUFA (165–288 mg (g TL)^−1^), mainly linoleic acid (C18:2n-6) and γ-linolenic acid (C18:3n-6)), and a low proportion of SFA (15–87 mg (g TL)^−1^). In comparison, the fatty acid composition of the biomass lipids and canola oil were quite similar (Figure 6i); the only difference was in the amounts of EPA (14–27 mg (g TL)^−1^) and DHA (38–79 mg (g TL)^−1^) (Figure 6j).

## 3. Discussion

### 3.1. Metabolism of Yeast Extract Nitrogen and Amino Acids in RT2316-16

Yeast extract is a widely used source of organic nitrogen in diverse culture media [12,13]. Yeast extract is relatively low in nitrogen (~11% w w^−1^ total nitrogen; ~7% w w^−1^ amino nitrogen; BD Bionutrients Technical Manual), but it provides all 20 amino acids (≈66% w w^−1^), both as free amino acids and in the form of peptides. These amino acids can directly contribute to protein synthesis in the microorganisms being grown. Compared to inorganic nitrogen, providing amino acids and peptides often improves microbial growth rate and biomass yield on carbon. The synthesis of proteins from inorganic nitrogen requires more energy compared to synthesis from already formed amino acid which explains the superior nutritional value of the yeast extract. In addition, amino acids are metabolized to precursors that contribute to the synthesis of other important metabolites (glucose and fats) that can be used to generate adenosine triphosphate (ATP) in the tricarboxylic acid cycle (TCA). Other nitrogen compounds in yeast extract include molecules such as guanine and guanosine-5’-triphosphate (GTP), vitamins (many B group vitamins), and growth factors.

Yeast extracts has been specifically identified as a good nitrogen source for thraustochytrids [9,10] including the strain RT2316-16. RT2316-16 uses yeast extract as a source of both carbon and nitrogen, to support biomass growth (Figure 1). The results suggest that glutamate could totally replace yeast extract if provided at a concentration greater than 3.6 g L^−1^ (Figure 1).

Although some thraustochytrids have been grown using glutamate as a sole source of nitrogen [8,14], how they might metabolize other amino acids is not known. Therefore, this work evaluated the ability of RT2316-16 to thrive on all 20 amino acids individually in media containing amino-acid-free yeast nitrogen base (YNB). It was observed that growth is possible in all amino acids except cysteine. Certain amino acids (that is, lysine, alanine, threonine, glutamine, or glutamate) promoted growth more than was possible in the amino-acid-free control medium. The final biomass concentration in media composed of glucose and any of the five growth-promoting amino acids was higher than in the amino-acid-free control medium (Figure 2a). As the above identified five growth-promoting amino acids are known to be metabolized differently, their growth-promoting effects may have had different mechanisms. For example, lysine is converted to acetoacetyl-CoA and acetyl-CoA, compounds involved in the synthesis of citrate and production of ATP via the TCA cycle. Metabolism of alanine and threonine generates pyruvate, which is converted to acetyl-CoA through the action of pyruvate dehydrogenase. In addition, pyruvate is converted to oxaloacetate via the action of pyruvate carboxylase. Glutamine acts as a nitrogen donor in the synthesis of nucleotides, hexosamine, and nonessential amino acids [15]. Glutamine is also involved in the synthesis of glutathione and ATP. The mitochondrial glutaminase converts glutamine into glutamate used in production of α-ketoglutarate through the action of transaminase or glutamate dehydrogenase. α-Ketoglutarate is an intermediate in the TCA cycle.

The growth of RT2316-16 was not stimulated by the other 15 amino acids, compared with the control (Figure 2a). This may have been simply because an incubation period longer than the investigated 48 h was likely needed to induce the enzymes to effectively metabolize some of these amino acids. Alternatively, the other nitrogen compounds in YNB may have interfered with the uptake and metabolism of the slowly metabolized amino acids. The significant growth of RT2316-16 in the control experiment was consistent with its metabolic pathways that had been reconstructed from its annotated genome, suggesting that it is potentially able to synthesize all amino acids except arginine [16].

The growth of biomass and synthesis of lipids require glucose to be consumed. In experiments that used a single amino acid in the medium, there was a strong linear correlation between the glucose consumed and the lipid content (R^2^ = 0.660) in the lipid-free biomass, as well as the total lipid-free biomass produced (R^2^ = 0.650) (Figure 2b,c). The metabolism of at least some of the amino acids affected the consumption of glucose in RT2316-16. Glucose can be used to synthesize lipids and therefore a high consumption of glucose correlated with a high lipid content in media that contained alanine, glutamine, glutamate, or serine. In the absence of amino acids in the medium, around 13.8% of the glucose was consumed, and the lipid content of the biomass was only 10.9% (w w^−1^).

The SFA content was similar in the total lipids of the biomass grown with alanine, glutamine, lysine, threonine, or glutamate. Moreover, the SFA in total lipids was comparable to that of the control biomass grown without amino acids. However, if none of the above-specified amino acids were used, the MUFA and the long-chain omega-3 PUFA in the total lipids of RT2316-16 were affected: the lipids contained more MUFA (43% higher than the average content in the lipids from the biomass grown with the amino acids) and fewer omega-3 PUFA compared to the lipids in the biomass grown with at least one of the specified amino acids. In comparison with the culture on glutamate, RT2316-16 grown with lysine, alanine, or serine produced lipids with a higher content of omega-3 PUFA.

### 3.2. Organic Nitrogen Promoted Mobilization of the Accumulated Lipids in RT2316-16

It was observed that, culture experiments conducted with different initial concentrations of glucose and the organic nitrogen (yeast extract–glutamate; measured as amino acids), showed a rapid initial growth phase of the lipid-free biomass. Subsequently, the growth rate declined as the concentration of amino acids in the medium decreased, and was completely arrested once these key nutrients were exhausted. During the first few hours of rapid growth, the total lipids in lipid-free biomass declined to around 8% (dry basis). These changes in lipid content implied that on inoculation into a fresh medium with plentiful organic nitrogen available, the stored lipids were mobilized to support rapid growth. A motile zoospore stage is part of the life cycle of many thraustochytrids. Zoospores are generally seen upon transfer to a fresh medium. In *Aurantiochytrium limacinum*, zoospores were observed upon transfer of grown cells to a fresh nutrient-poor medium but not to a medium containing yeast extract [17]. In zoospores, various genes coding for lipases (which contribute to the hydrolysis of ester bonds of triacylglycerols and diacylglycerols) are up-regulated, and the released fatty acids are conveyed preferentially to the peroxisome to be oxidized [17]. The loss of stored lipids occurred concurrently with the consumption of the carbon source (glucose or glycerol). Experiments with canola oil showed that RT2316-16 could utilize triacylglycerols in the medium as a carbon source for growth and lipid synthesis.

After the initial consumption of stored lipids, a lipid accumulation phase began and lasted until glucose (or glycerol) remained in the culture medium. The exhaustion of glucose promoted lipid mobilization if organic nitrogen was available. In contrast, lipids continued to accumulate if organic nitrogen was totally consumed but glucose remained. The lowering of the total lipids in the biomass towards the end (a period of low organic nitrogen in the medium) of the cultures grown in glycerol and canola oil did not have a clear explanation, but lipids may have been consumed via β-oxidation to meet metabolic needs.

For otherwise similar conditions, the type of the carbon source (whether glucose, glycerol, or canola oil) had little effect on the final lipid-free biomass concentration and the growth rate of the lipid-free biomass (r_lfb_ = 0.07–0.09 g (L h)^−1^; Table 1). In experiments with the different carbon sources, the initial concentration of amino acids (provided by yeast extract—glutamate) in the medium was the same. In all these cases, organic nitrogen was the growth limiting substrate, and the carbon source was used to generate energy and lipids.

During the initial linear growth, the consumption of glucose and amino acids was linear with time (Table 1; Figure 3). The linear increase of the lipid-free biomass (as opposed to exponential growth) was likely associated with a continuous linear decline in the concentration of amino acids, the growth limiting substrate.

### 3.3. Carotenoid Synthesis and Possible Functions in RT2316-16

In batch cultures, the total carotenoids in the lipid-free biomass decreased during the early part of incubation with a simultaneous loss in the total lipids of the biomass. After this initial period, the carotenoid content increased, but in some cases, there was a loss in carotenoids towards the end of the culture. This concurred with earlier work in repetitive batch culture that had shown both the carotenoid content and composition to be influenced by the composition of the culture medium used to grow the biomass [6]. A medium rich in yeast extract promoted the accumulation of carotenoids in the biomass. In RT2316-16, the total carotenoids were substantially lowered if inorganic nitrogen (ammonium sulfate, ammonium chloride, or sodium nitrate) was used for the culture (not shown). Some of the vitamins, or other metabolites, in yeast extract can therefore be concluded to enhance carotenoids synthesis compared to culture on inorganic nitrogen.

In RT2316-16, the synthesis of isopentenyl diphosphate, the 5-carbon precursor of isoprenoids and carotenoids, occurs in the mevalonic acid pathway (MVA) (Figure S1 in Supplemental Material) [16]. This pathway starts with acetyl-CoA and acetoacetyl-CoA, which are produced from glucose or the metabolism of some amino acids. The first reaction in the MVA pathway is catalyzed by hydroxymethylglutaryl-CoA synthase (HMG-CoA synthase), producing (S)-3-hydroxy-3-methylglutaryl-CoA (HMG-CoA). In the next step, HMG-CoA reductase transforms HMG-CoA into MVA. HMG-CoA synthase and HMG-CoA reductase are both strongly regulated [18,19]. The 5’-adenosine monophosphate-activated protein kinase (AMPK), an energy sensor that is activated by an increase in the AMP/ATP ratio (low energy), regulates the activity of HMG-CoA reductase through its phosphorylation [20]. Although carotenoids are not the main metabolites in the MVA pathway, the growth promoting effect of yeast extract under energy sufficiency would ensure an adequately high carbon flux through this pathway so that some isopentenyl diphosphate could be diverted to the synthesis of carotenoids. At present, it is uncertain whether carotenoid synthesis (or the flux through the MVA pathway) requires a particular amino acid, or amino acid derivative, or if it is determined solely by the magnitude of the flux through the MVA pathway under culture conditions that promote growth of the lipid-free biomass.

The observed loss of total lipids and carotenoids during early incubation was nearly simultaneous (Figure 3 and Figure 4), suggesting that these processes were somehow linked. The main carotenoid produced by RT2316-16 is β-carotene [6], a lipophilic molecule that is apportioned between the cell membranes and the lipid droplets. The initial loss of total carotenoids may have been due to β-oxidation of fatty acids, that lowers the total quantity (number and size) of lipid droplets.

Lipid reserves are mobilized through β-oxidation of fatty acids that occurs in the mitochondria or peroxisomes [21,22]. The specifics of β-oxidation in RT2316-16 are not known. In the mitochondrial β-oxidation, the acyl-CoA is oxidized producing enoyl-CoA and FADH2 equivalents that can transfer electrons directly to the electron transfer chain for producing ATP. The enzymes of the electron transfer chain are major sources of reactive oxygen species (ROS). Peroxisomal β-oxidation uses alternative oxidases that reduce molecular oxygen to hydrogen peroxide, a ROS, without the formation of FADH2. In order to mitigate oxidative damage, the level of ROS within the cell is controlled by both enzymatic and non-enzymatic mechanisms. Carotenoids quench ROS non-enzymatically.

The observed changes in total carotenoids and total lipids with the changes in availability of carbon and organic nitrogen suggest a possible regulatory role of carotenoids in lipid metabolism (lipogenesis and fatty acid oxidation) in RT2316-16. In studies with mammalian adipocytes, treatment with β-carotene has been found to lower the cellular content of triacylglycerols and increase the oxidation of fatty acids [23]. This adipogenic effect is apparently due to retinoic acid and retinoids produced from all-trans-retinal. The latter is a product of the oxidative cleavage of β-carotene, a reaction catalyzed by β,β-carotene 15,15’-monooxygenase-1 [24,25]. The gene sequence Thraus_T2025 coding for β,β-carotene 15,15’-monooxygenase-1 (BCMO1) (BCDO1_HUMAN Swissprot database) has been reported in RT2316-16 [16].

### 3.4. EPA, DPA and DHA Accumulated during Consumption of the Accumulated Fatty Acids in RT2316-16

The fatty acid composition and content of the total lipids of RT2316-16 depended on the composition of the culture medium and the incubation period (Figure 6). Significantly, a high content of omega-3 PUFA (EPA, DPA, and DHA) in the total lipids could be obtained by growing RT2316-16 in a medium with a low glucose concentration (6 g L^−1^) in combination with a high concentration of organic nitrogen (yeast extract 12 g L^−1^; glutamate 1.06 g L^−1^) (Figure 4d). The production of DHA by *Crypthecodinium cohnii*, a heterotrophic marine dinoflagellate, was negatively affected by feeding yeast extract. However, the proper feeding with concentrated yeast extract solution did increase DHA productivity [26]. PUFA were specifically elevated after the exhaustion of glucose and the subsequent mobilization of total lipids. Increases in the DHA and EPA content were also observed during lipid mobilization at the end of the incubation period in the MH medium and also with glycerol as the carbon source. These results might suggest that the activity of desaturases, involved in the omega-3 PUFA synthesis, and fatty acid β-oxidation are related through some unknown mechanism in RT2316-16. In mammalian cells, PUFA affect the expression of genes encoding the proteins involved in fatty acid oxidation while simultaneously downregulating genes encoding proteins for lipid synthesis [27].

While EPA and DHA occurred in the total lipids of the biomass grown in all the media tested, it was observed that DPA occurred only in the biomass grown in the ML medium. The omega-6 (n-6) DPA has also been reported in thraustochytrids such as *Aurantiochytrium limacinum* and *Thraustochytrium aureum* [4]. In contrast, in *Parietichytrium* sp., which uses only the elongase–desaturase pathway for PUFA synthesis, the omega-3 form of DPA (n-3 DPA) is an intermediate between EPA and DHA in the pathway [4].

The carbon source had a substantial effect on the composition of the fatty acids in RT2316-16. While glucose metabolism results in lipids with high levels of MUFA and PUFA, the use of glycerol as the carbon source favors the production of SFA and MUFA. The similar fatty acid profiles of canola oil and the microbial lipids in the biomass grown in this oil implied that the fatty acids derived from canola oil were directly incorporated in the microbial glycerolipids. Radiolabeled oleic acid, linoleic acid and α-linolenic acid accumulated directly into glycerolipids in *Thraustochytrium* sp. 26185 [28].

## 4. Materials and Methods

### 4.1. Culture Experiments

*Inoculum preparation*. *Thraustochytrium* sp. RT2316-16 [6,16] was used in all experiments. The stock cultures were stored in glycerol (50% *v/v*) at −18 °C. experiments were carried out aseptically. The inocula for all experiments was prepared as follows: A 250 mL Erlenmeyer flask containing 100 mL of sterile medium M was inoculated with 1 mL of the pure stock culture and incubated on an orbital shaker (150 rpm, 15 °C) for 5 days. All cultures were incubated in the dark. A 5 mL portion of the grown culture was used to inoculate 100 mL of fresh, sterile medium. This second culture was the inoculum for the experiments. The medium was sterilized by autoclaving (121 °C, 20 min).

*Effect of the components in the control medium (M) on the production of biomass and lipids*. The medium M contained the following components (per L of medium): glucose (Gl) (Merck KGaA, Darmstadt, Germany) 20 g, yeast extract (YE) (Merck, Darmstadt, Germany) 6 g, and monosodium glutamate (E) (Merck, Darmstadt, Germany) 0.6 g, in half-strength artificial seawater (ASW) [9]. Cultures were carried out using the following combinations of components: Gl-YE-E, Gl-YE, YE-E, Gl-E. The concentration of each component was the same as in medium M. Glucose with E at 2.4 g L^−1^ (Gl-E*) and 3.6 g L^−1^ (Gl-E**), was also evaluated. The inoculum for these cultures was prepared in medium M. The culture experiments were carried out in Erlenmeyer flasks (250 mL; 100 mL of the respective medium per flask) and incubated for 5 days on an orbital shaker (150 rpm, 15 °C). Each medium was evaluated in triplicate. The biomass was recovered by centrifugation (2057× *g* 10 min, 4 °C), washed with distilled water, freeze-dried, weighed, and stored at −20 °C until further analysis. A portion of the culture supernatant was filtered (0.2 μm nominal pore size polytetrafluoroethylene (PTFE) membrane) and frozen (−20 °C) until further analysis.

*Effect of amino acids on the production of biomass and lipids*. The inoculum for this set of experiments was prepared in the M medium. After 5 days incubation, the biomass was recovered by centrifugation (2057× *g* 10 min, 4 °C), washed twice with sterile and diluted ASW, and suspended in sterile and diluted ASW. The Erlenmeyer flasks (125 mL, containing 50 mL of sterile medium each) were inoculated with 5 mL of the washed biomass. The sterile medium contained: yeast nitrogen base (YNB) without amino acids (BD Difco^TM^; Sparks, NV, USA), dissolved in sterile and diluted ASW, filtrated using a 0.2 μm membrane) 6.7 g L^−1^, glucose 5 g L^−1^, and the specified amino acid 1 g L^−1^ in diluted ASW. All the 20 amino acids were from Sigma (St. Louis, MO, USA). The incubation period was 2 days (150 rpm, 15 °C). Each medium was evaluated in triplicate. The biomass was recovered by centrifugation (2057× *g* 10 min, 4 °C), washed with distilled water, freeze-dried, weighed, and stored at −20 °C until further analysis. A portion of the culture supernatant was filtered (0.2 μm nominal pore size polytetrafluoroethylene (PTFE) membrane) and frozen (−20 °C) until further analysis.

*Effect of glucose and yeast extract concentration on the production of biomass and lipids*. The inoculum for each growth curve was prepared in the tested medium. The compositions of the tested media were: glucose 20 g L^−1^, yeast extract 6 g L^−1^, monosodium glutamate 0.6 g L^−1^ (medium M); glucose 6 g L^−1^, yeast extract 12 g L^−1^, monosodium glutamate 1.2 g L^−1^ (medium ML); glucose 20 g L^−1^, yeast extract 12 g L^−1^, monosodium glutamate 1.2 g L^−1^ (medium MH). Glycerol and canola oil were tested separately as carbon sources, each at 20 g L^−1^ (medium M without glucose). The twenty-four Erlenmeyer flasks (250 mL, containing 100 mL of sterile medium) were inoculated with 5 mL of the grown culture. The incubation was at 150 rpm and 15 °C. Every 12 or 24 h, three flasks were sacrificed, and the biomass was recovered by centrifugation (2057× *g* 10 min, 4 °C). The biomass was washed with distilled water, freeze-dried, weighed, and stored at −20 °C until further analysis. A portion of the culture supernatant was filtered (0.2 μm nominal pore size PTFE membrane) and frozen (−20 °C) until further analysis.

### 4.2. Analyses

*Concentrations of biomass and residual sugars*. Concentration of biomass (dry weight, DW) was measured gravimetrically by recovering the cells by centrifugation (7000× *g*, 4 °C, 10 min) from a known volume of culture (~10 mL). The cell pellet was washed twice with distilled water (5 mL per wash), recovered by centrifugation, and dried (65 °C) to a constant weight. Concentrations of residual glucose and glycerol were measured by high-performance liquid chromatography (HPLC) [6].

*Concentration of ammonium*. Ammonium concentration in the culture supernatant was determined spectrophotometrically [7]. The supernatant sample (100 µL) was mixed with 1 mL of o-phthaldehyde (OPA; Sigma) reagent (5 mg OPA dissolved in 100 µL of pure ethanol, 5 µL of β-2-mercaptoethanol and 10 mL of 50 mM carbonate buffer, pH 10.5) and the absorbance was measured at 340 nm exactly 2 min after mixing. The blank was made as above, but with the sample replaced by half-strength ASW. The standard solutions of L-lysine (Sigma, St. Louis, MO, USA) in diluted ASW were used to make the calibration curve.

*Extraction of total lipids and determination of the fatty acid profile*. Total lipids (TL) in the biomass were extracted using the Bligh and Dyer method [29]. A 50 mg portion of the freeze-dried biomass was extracted (1 h, 150 rpm) with 9.5 mL of a solvent mixture of chloroform, methanol, and phosphate buffer (50 mM, pH 7.4) 2.5: 5.0: 2.0 by volume. This slurry was transferred to a separating funnel containing 2.5 mL of chloroform. After mixing, 2.5 mL of phosphate buffer was added, and the contents were mixed and allowed to separate. The chloroform layer was recovered, the solvent was evaporated at room temperature, and the residue of the extracted TL was weighed. Additionally, the conditions for methylation and analysis of the fatty acid methyl esters were the same as previously described [6].

*Extraction and quantification of total carotenoids*. A culture aliquot (3–5 mL) was centrifuged (2057× *g*, 10 min) and the supernatant was discarded. The carotenoid extraction and quantification followed published methods [6].

*Thin-layer chromatography*. The lipid extracts (Bligh and Dyer methodology [29]) were diluted in chloroform at a concentration of 10 mg mL^−1^. The separation and identification of neutral lipids were carried out according to the method described by Kishimoto et al. [30]. The aluminum TLC plate (silica gel 60 F254; Merck) was washed with the mobile phase hexane/diethyl ether/acetic acid (80/30/1, *v/v/v*) and dried at room temperature; 9 μL of lipids in chloroform were loaded. The TLC plate was developed with iodine vapor.

*Statistical analysis*. MATLAB (MathWorks, Inc., Natick, MA, USA) was used to perform one-way analysis of variance (ANOVA) and comparison of the means at a 95% confidence level.

## 5. Conclusions

The growth and lipid synthesis in the marine thraustochytrid RT2316-16 were affected by the amino acids available in the culture medium. Alanine, glutamine, lysine, threonine, and serine that were supplied individually promoted the production of lipids that contained long-chain omega-3 PUFA when the initial glucose concentration was 5 g L^−1^. When the thraustochytrid was grown with yeast extract as a source of amino acids, the biomass with the highest content of EPA and DHA was produced under the following conditions: glucose 5 g L^−1^, yeast extract 12 g L^−1^ and monosodium glutamate 1.2 gL^−1^. The results suggest that the long-chain omega-3 PUFA content of other thraustochytrids may be influenced by the composition of the culture medium and the provision of specific amino acids in the medium. Cultivation of RT2316-16 and similar strains, should consider harvest after glucose is exhausted to obtain biomass with a high content of omega-3 PUFA in the lipids, although there may be a loss of total lipids in the biomass.

## Figures and Tables

**Table 1:** Kinetic parameters of growth of *Thraustochytrium* sp. RT2316-16 in batch culture with different media compositions: growth rate of the lipid-free biomass (Xlf) r_xlf_, consumption rate of the carbon source (glucose or glycerol) –r_S_, consumption rate of amino acids (from yeast extract and glutamate) –r_AA_, accumulation rate of total lipids (TL) r_TL_ in lipid-free biomass, and accumulation rate of total carotenoids (TC) r_TC_ in lipid-free biomass. The coefficients of determination (R^2^) for various linear relationships are also shown.

Growth Medium *	C-Source		N-Source		Xlf	TC		TL	
	−r_S_	R^2^	−r_AA_×10	R^2^	r_Xlf_	r_TC_	R^2^	r_TL_	R^2^
M	0.17	0.978	0.78	0.955	0.09	1.30	0.953	4.07	0.966
	0.06	0.942						1.22	0.963
ML	0.08	0.960	0.84	0.944	0.12	1.25	0.961	3.16	0.983
			0.29	0.967		−0.87	0.945	−1.15	0.922
MH	0.13	0.989	1.55	0.999	0.20	2.77	1.000	4.04	1.000
			0.31	0.979		0.42	0.904	0.45	0.777
M-CO	n.d.	n.d.	0.94	0.797	0.07	1.22	0.960	0.0	
			0.16	0.967				5.24	0.995
M-Glycerol	0.08	0.977	0.68	0.96	0.07	1.84	0.757	5.00	0.962
	0.02	0.996			0.05	0.0		13.08	0.995

* Initial concentration of glucose, yeast extract, and monosodium glutamate: 20, 6, and 0.6 g L^−1^ (medium M); 6, 12, and 1.2 g L^−1^ (medium ML); 20, 12, and 1.2 g L^−1^ (medium MH). M-CO as medium M with glucose replaced by canola oil. M-Glycerol as medium M, with glucose replaced by glycerol. Units of the rates (r_S_, r_NH4_, r_Xlf_) are g L^−1^ h^−1^; units of r_TC_ and r_TL_ are μg (g of lipid-free biomass)^−1^ h^−1^ and mg (g of lipid-free biomass)^−1^ h^−1^, respectively. n.d.: not determined.

## Data Availability

Not applicable.

## Supplementary Materials

The following supporting information can be downloaded at: https://www.mdpi.com/article/10.3390/md21010015/s1: Figure S1: Terpenoid backbone biosynthesis in *Thraustochytrium* sp. RT2316-16. The results were obtained with the KEGG Mapper Reconstruction Tool. Red boxes denote enzymes coded by the genes annotated in the genome [16]. Table S1: Effect of the individual amino acids on the final concentrations of total biomass and glucose, glucose consumption, and total lipid content of the biomass of *Thraustochytrium* sp. RT2316-16 grown at 15 °C for 48 h. Table S2: Effect of the individual amino acids (alanine A, glutamine Q, lysine K, threonine T, glutamate E, serine S) on the fatty acid composition of the total lipids in the biomass of *Thraustochytrium* sp. RT2316-16 grown at 15 °C for 48 h. The control medium corresponds to the lipids in the biomass grown without amino acids. Table S3: Amino acid composition of the yeast extract used in media for growing *Thraustochytrium* sp. RT2316-16. Table S4: Fatty acid composition of total lipids in the biomass of *Thraustochytrium* sp. RT2316-16 at various times during incubation. The biomass was grown at 15 °C in medium M (glucose 20 g L^−1^, yeast extract 6 g L^−1^, monosodium glutamate 0.6 g L^−1^). Table S5: Fatty acid composition of total lipids in the biomass of *Thraustochytrium* sp. RT2316-16 at various times during incubation. The biomass was grown at 15 °C in medium ML (glucose 5 g L^−1^, yeast extract 12 g L^−1^, monosodium glutamate 1.2 g L^−1^). Table S6: Fatty acid composition of total lipids in the biomass of *Thraustochytrium* sp. RT2316-16 at various times during incubation. The biomass was grown at 15 °C in medium MH (glucose 20 g L^−1^, yeast extract 12 g L^−1^, monosodium glutamate 1.2 g L^−1^). Table S7: Fatty acid composition of total lipids in the biomass of *Thraustochytrium* sp. RT2316-16 at various times during incubation.The biomass was grown at 15 °C in medium M with glycerol as the carbon source. Table S8: Fatty acid composition of total lipids in the biomass of *Thraustochytrium* sp. RT2316-16 at various times during incubation. The biomass was grown at 15 °C in medium M with canola oil as the carbon source.
