# Supplementary material for: Nitrogen Sources Affect the Long-Chain Polyunsaturated Fatty Acids Content in Thraustochytrium sp. RT2316-16
Source: Mar Drugs. 2022 Dec 25;21(1):15. doi: 10.3390/md21010015 (PMC9864179; doi:10.3390/md21010015)
Supplement: Supplementary file 1 [file marinedrugs-21-00015-s001.zip › marinedrugs-2113484-supplementary.pdf]

SUPPLEMENTAL MATERIAL

Nitrogen sources affect the long-chain polyunsaturated fatty acids content in *Thraustochytrium* sp. RT2316-16

Diego Valdebenito[1], Sebastián Urrutia[1], Allison Leyton[1], Yusuf Chisti[2], Juan A. Asenjo[3] and Carolina Shene[1,*]

[1]Center for Biotechnology and Bioengineering (CeBiB), Center of Food Biotechnology and Bioseparations, BIOREN and Department of Chemical Engineering, Universidad de La Frontera, Francisco Salazar 01145, Temuco 4780000, Chile

[2]Institute of Tropical Aquaculture and Fisheries, Universiti Malaysia Terengganu, Kuala Nerus 21030, Terengganu, Malaysia

[3]Centre for Biotechnology and Bioengineering (CeBiB), Department of Chemical Engineering and Bio-technology, Universidad de Chile, Beauchef 851, Santiago 8370459, Chile

[*]Correspondence: carolina.shene@ufrontera.cl

Figure S1: Terpenoid backbone biosynthesis in *Thraustochytrium* sp. RT2316-16. Results were obtained with the KEGG Mapper Reconstruction tool. Red boxes denote enzymes coded by the genes annotated in the genome [16].

Table S1: Effect of the individual amino acid on the final concentrations of total biomass and glucose, glucose consumption and total lipid content of the biomass of *Thraustochytrium* sp. RT2316-16 grown at 15 °C for 48 h.

Table S2: Effect of the individual amino acid (alanine A, glutamine Q, lysine K, threonine T, glutamate E, serine S) on fatty acid composition of the total lipids in the biomass of *Thraustochytrium* sp. RT2316-16 grown at 15 °C for 48 h. Control corresponds to the lipids in the biomass grown without amino acids.

Table S3: Amino acid composition of the yeast extract used in media for growing *Thraustochytrium* sp. RT2316-16.

Table S4: Fatty acid composition of total lipids in the biomass of *Thraustochytrium* sp. RT2316-16 at various times during incubation. The biomass was grown at 15 °C in medium M (glucose 20 g L^{-1}, yeast extract 6 g L^{-1}, monosodium glutamate 0.6 g L^{-1}).

Table S5: Fatty acid composition of total lipids in the biomass of *Thraustochytrium* sp. RT2316-16 at various times during incubation. The biomass was grown at 15 °C in medium ML (glucose 5 g L^{-1}, yeast extract 12 g L^{-1}, monosodium glutamate 1.2 g L^{-1}).

Table S6: Fatty acid composition of total lipids in the biomass of *Thraustochytrium* sp. RT2316-16 at various times during incubation. The biomass was grown at 15 °C in medium MH (glucose 20 g L^{-1}, yeast extract 12 g L^{-1}, monosodium glutamate 1.2 g L^{-1}).

Table S7: Fatty acid composition of total lipids in the biomass of *Thraustochytrium* sp. RT2316-16 at various times during incubation. The biomass was grown at 15 °C in medium M with glycerol as the carbon source.

Table S8: Fatty acid composition of total lipids in the biomass of *Thraustochytrium* sp. RT2316-16 at various times during incubation. The biomass was grown at 15 °C in medium M with canola oil as the carbon source.

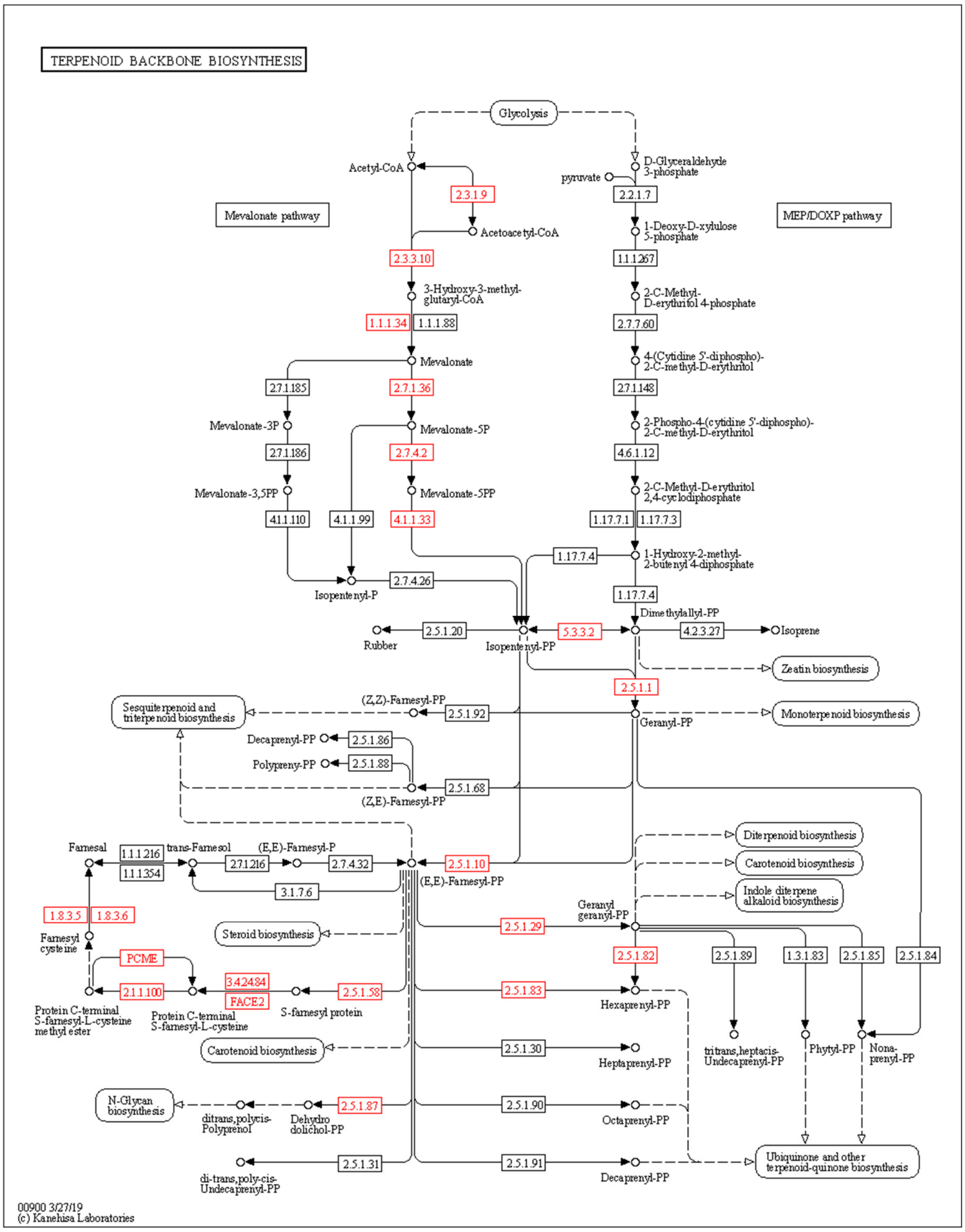

Figure S1: Terpenoid backbone biosynthesis in *Thraustochytrium* sp. RT2316-16. Results were obtained with the KEGG Mapper Reconstruction tool. Red boxes denote enzymes coded by the genes annotated in the genome [16].

Table S1: Effect of the individual amino acid on the final concentrations of total biomass and glucose, glucose consumption and total lipid content of the biomass of *Thraustochytrium* sp. RT2316-16 grown at 15 °C for 48 h.

	Biomass (g L^{-1})	Final glucose (g L^{-1})	Glucose consumption (g L^{-1})	Lipid content (% DW)
Alanine	2.15	2.2	56.2	18.1
Glutamine	2.05	0.6	88.0	19.7
Lysine	1.70	2.2	56.7	11.7
Threonine	1.65	2.8	43.9	12.8
Glutamate	1.60	1.7	66.1	20.5
Control (without amino acids)	1.50	4.3	13.8	10.9
Glycine	1.45	2.5	50.2	15.0
Serine	1.30	2.2	56.7	21.6
Asparagine	1.25	2.4	53.0	15.2
Leucine	1.20	2.5	50.8	12.8
Arginine	1.15	3.6	28.0	12.1
Phenylalanine	1.10	3.5	29.0	16.8
Proline	1.10	3.8	24.8	8.4
Tyrosine	1.05	3.2	35.1	11.5
Histidine	0.95	4.2	16.4	6.8
Isoleucine	0.95	3.8	23.8	9.4
Tryptophan	0.85	3.8	24.8	7.9
Methionine	0.85	4.1	18.5	8.6
Valine	0.70	3.8	24.1	14.5
Aspartic acid	0.55	4.5	10.3	7.3
Cysteine	0.35	4.6	7.1	6.6

Table S2: Effect of the individual amino acid (alanine A, glutamine Q, lysine K, threonine T, glutamate E, Serine S) on fatty acid composition of the total lipids in the biomass of *Thraustochytrium* sp. RT2316-16 grown at 15 °C for 48 h. Control corresponds to the lipids in the biomass grown without amino acids.

FA	A	Q	K	T	E	S	Control
C12:0	4.7 ± 0.2	3.4 ± 0.7	8.2 ± 1.9	5.7 ± 0.3	5.5 ± 0.8	5.4 ± 0.0	8.4 ± 1.8
C14:0	30.7 ± 1.4	27.3 ± 5.6	32.7 ±5.0	29.6 ± 1.1	37.9 ± 5.6	32.5 ± 1.3	46.3 ± 6.6
C14:1	n.d.	n.d.	3.3 ± 1.0	1.9 ± 0.1	n.d.	2.0 ± 0.0	n.d.
C16:0	138.1 ± 8.1	139.0 ± 10.0	127.6 ± 9.0	132.1 ± 5.6	151.4 ± 19.3	136.0 ± 7.6	159.9 ± 16.0
C16:1	32.8 ± 1.8	31.9 ± 6.3	29.9 ± 5.0	37.4 ± 1.3	50.8 ± 6.9	38.8 ± 2.2	80.0 ± 5.5
C18:0	36.4 ± 2.6	43.5 ± 2.1	59.1 ± 5.0	31.0 ± 1.3	35.8 ± 5.0	36.2 ± 0.8	52.2 ± 13.9
C18:1n-9	112.3 ± 6.3	99.9 ± 10.0	116.2 ± 9.0	110.6 ± 1.3	98.5 ± 14.8	111.6 ± 3.2	136.6 ± 11.0
C18:2n-6	38.0 ± 2.0	32.6 ± 2.0	33.0 ± 2.0	29.9 ± 1.2	30.9 ± 5.0	36.5 ± 0.9	33.1 ± 4.4
C18:3n-6	11.2 ± 0.5	9.8 ± 2.0	9.5 ± 4.1	9.2 ± 0.7	7.9 ± 1.0	10.5 ± 0.5	7.1 ± 0.1
C20:2	n.d.	n.d.	n.d.	n.d.	n.d.	1.4 ± 0.0	n.d.
C20:3n-6	4.2 ± 0.2	3.2 ± 0.9	n.d.	2.8 ± 0.2	2.6 ± 0.5	3.9 ± 0.2	n.d.
C20:4	6.5 ± 0.0	4.6 ± 1.0	6.8 ± 3.3	5.2 ± 0.4	3.7 ± 0.7	5.9 ± 0.2	2.6 ± 0.3
C24:0	2.9 ± 0.4	2.5 ± 0.6	n.d.	2.3 ± 0.1	2.4 ± 0.8	2.7 ± 0.1	n.d.
C20:5n-3	11.7 ± 0.2	9.4 ± 1.3	14.2 ± 6.8	10.5 ± 0.9	6.3 ± 0.9	10.5 ± 0.5	3.6 ± 1.2
C24:1	9.7 ± 0.7	5.9 ± 1.7	10.0 ± 5.0	11.7 ± 0.9	5.0 ± 1.1	8.6 ± 0.3	4.0 ± 0.1
C22:5n-3	15.1 ± 0.8	10.1 ± 2.4	18.3 ± 3.0	18.5 ± 1.2	8.8 ± 1.8	13.7 ± 0.6	6.0 ± 0.5
C22:6n-3	22.0 ± 1.6	19.3 ± 1.3	33.3 ± 2.0	19.7 ± 2.1	10.3 ± 0.9	18.7 ± 1.5	4.7 ± 2.6
Total	476	442	502	458	458	475	544
SFA	212.9 ± 12.8	215.6 ± 18.9	227.6 ± 20.9	200.6 ± 8.4	233.1 ± 31.5	212.7 ± 9.9	266.7 ± 38.3
MUFA	154.8 ± 8.8	137.7 ± 18.0	159.4 ± 20.0	161.6 ± 3.5	154.2 ± 22.8	161.0 ± 5.7	220.7 ± 16.6
PUFA	108.6 ± 5.3	89.0 ± 11.0	115.0 ± 21.2	95.9 ± 6.7	70.4 ± 10.8	101.1 ± 4.3	57.1 ± 9.0

w3	33.7 ± 1.8	28.7 ± 2.7	47.5 ± 8.8	30.2 ± 3.0	16.6 ± 1.8	29.2 ± 2.0	8.3 ± 3.8
w6	59.8 ± 3.5	50.2 ± 8.3	49.2 ± 12.5	47.2 ± 3.7	45.0 ± 9.0	56.8 ± 2.3	42.8 ± 5.2

n.d.: not detected. All data are in mg (g TL)$^{-1}$).

Table S3: Amino acid composition of the yeast extract used in media for growing *Thraustochytrium* sp. RT2316-16.

Amino acid	Content ($mg\ g^{-1}$)	(w/w %)
Aspartic	30.2 ± 2.6	3.0
Serine	9.7 ± 3.7	1.0
Glutamate	56.2 ± 5.3	5.6
Glycine	44.7 ± 3.4	4.5
Histidine	20.9 ± 0.4	2.1
Arginine	38.9 ± 0.6	3.9
Threonine	35.0 ± 0.4	3.5
Alanine	55.0 ± 0.2	5.5
Proline	30.0 ± 0.8	3.0
Cysteine	1.3 ± 0.1	0.1
Tyrosine	11.7 ± 0.7	1.2
Valine	51.1 ± 0.7	5.1
Methionine	14.5 ± 0.1	1.5
Lysine	56.8 ± 0.2	5.7
Isoleucine	40.2 ± 0.3	4.0
Leucine	55.1 ± 0.4	5.5
Phenylalanine	26.1 ± 1.3	2.6

Methodology. A 100 mg sample of yeast extract was placed in a glass test tube and 5 mL of 6 M HCl were added. The tube was flushed with nitrogen, capped, and placed in a heating block at 112 °C for 22 h. The tube was cooled, an internal standard (10 mL of 2.5 mM α-aminobutyric acid) was added to the hydrolysate, and the resulting solution was made up to 250 mL with MilliQ® water in a volumetric flask. A 0.5 mL portion of this solution was filtered through a 0.45 μm PTFE membrane for further use. The hydrolyzed sample and standards were treated following the instructions that accompanied the Waters AccQ-Tag™ Ultra Derivatization Kit (Waters Inc., Milford, MA, USA). The prepared samples were analyzed by HPLC (C18, 4 μm, 3.9×150 mm column; Waters Inc., Milford, MA, USA). The column temperature was 37 °C. A UV detector (248 nm) was used (Waters Inc., Milford, MA, USA). Elution rate was constant at 1 mL min^{-1}. Mobile phases consisted of (A) 100% AccQ-Tag Eluent A concentrate, (B) 100% acetonitrile HPLC-grade (Merck), and (C) 100% HPLC-grade water. A gradient elution was used, as follows: 0–0.5 min 100% A; 0.5–18 min 99% A and 1% B; 18–19 min 95% A and 5% B; 19–28 min 91% A and 9% B; 28–35 min 83% A and 17% B; 35–38 min 60% B and 40% C; and 38–40 min 100% A.

Table S4: Fatty acid composition of total lipids in the biomass of *Thraustochytrium* sp. RT2316-16 at various times during incubation. The biomass was grown at 15 °C in medium M (glucose 20 g L^{-1}, yeast extract 6 g L^{-1}, monosodium glutamate 0.6 g L^{-1}).

Fatty acids	12 h	24 h	48 h	72 h	96 h	120 h	144 h	168 h
	(mg (g TL)$^{-1}$)							
C10:0	n.d.	n.d.	n.d.	3.6 ± 0	4.6 ± 0.6	6.3 ± 0.3	3.7 ± 0.1	n.d.
C12:0	n.d.	n.d.	7.6 ± 0.8	7.5 ± 0.4	12.2 ± 1.2	17.4 ± 0.1	10.9 ± 0.1	8.6 ± 2.7
C14:0	n.d.	7.1 ± 1.6	18.8 ± 2.3	24.8 ± 2.3	41.9 ± 6.8	43 ± 1.0	36.3 ± 4.4	30.6 ± 2.0
C14:1	n.d.	n.d.	n.d.	3.4 ± 0.4	4.5 ± 1.5	5.4 ± 0.2	3.5 ± 0.1	3.3 ± 0.3
C16:0	84.2 ± 8.8	56.1 ± 10.4	34.2 ± 1.0	65.9 ± 12.6	119.9 ± 53.6	75.1 ± 3.2	121.3 ± 25.2	123.9 ± 10.0
C16:1	26.5 ± 3.9	7.5 ± 1.7	n.d.	6.0 ± 0.2	5.5 ± 1	18.9 ± 2.3	33.3 ± 10.9	25.7 ± 6.0
C17:1	n.d.	n.d.	n.d.	n.d.	n.d.	2.8 ± 0.1	n.d.	n.d.
C18:0	46.9 ± 2.3	13.4 ± 1.9	61.6 ± 5.8	33.7 ± 4.6	166 ± 21.9	111.2 ± 9.6	68 ± 1	69.8 ± 6.4
C18:1n-9	65.4 ± 6.5	64.2 ± 20.4	54.7 ± 7.8	94.9 ± 18.1	104.8 ± 1.6	122.3 ± 7.3	144.6 ± 23.4	167.8 ± 3.1
C18:2n-6	29.3 ± 4.0	25.3 ± 7.1	4.6 ± 0.5	29.8 ± 4.6	18.1 ± 13	53.3 ± 10.4	60.7 ± 5.2	52.0 ± 10
C18:3n-6	n.d.	n.d.	n.d.	2.4 ± 0.3	n.d.	n.d.	5.5 ± 1.5	4.0 ± 1.4
C20:1	n.d.	n.d.	n.d.	n.d.	n.d.	n.d.	n.d.	n.d.
C18:3n-3	n.d.	n.d.	n.d.	n.d.	n.d.	n.d.	n.d.	n.d.
C20:2	n.d.	n.d.	n.d.	n.d.	n.d.	4.3 ± 0.7	4.5 ± 0.3	4.1 ± 2.0
C20:3n-6	n.d.	n.d.	n.d.	n.d.	n.d.	3.7 ± 0.2	5.0 ± 0.6	3.8 ± 1.7
C22:1	n.d.	n.d.	5.5 ± 2.6	6.9 ± 0.9	9 ± 1.5	20.8 ± 1.5	7.6 ± 1.4	4.7 ± 3.8
C20:4n-6	n.d.	7.8 ± 2.2	n.d.	9.7 ± 1.1	4.3 ± 1.2	6.0 ± 1.2	10.9 ± 1.6	8.5 ± 2.6
C23:0	9.0 ± 2.1	n.d.	n.d.	n.d.	n.d.	n.d.	n.d.	n.d.
C24:0	n.d.	n.d.	n.d.	n.d.	n.d.	1.9 ± 0.1	2.4 ± 0.1	3.1 ± 0.7

C20:5n-3	33.7 ± 6.2	37.2 ± 10.1	24.1 ± 1.0	28.1 ± 3.7	16.3 ± 1.6	19 ± 2.8	27.3 ± 3.7	27.2 ± 3.9
C24:1	n.d.	14.9 ± 4.2	7.3 ± 1.8	8.2 ± 1.2	6 ± 4.5	7.8 ± 0.3	13 ± 1.5	11.1 ± 3.2
C22:5n-3	n.d.	n.d.	n.d.	20.6 ± 3.8	23.9 ± 2.6	39.2 ± 2.7	42.2 ± 2.8	34.2 ± 11.8
C22:6n-3	104.9 ± 16	122.5 ± 33.1	95 ± 21.4	77.2 ± 12.2	55.1 ± 8	55.6 ± 3.1	71.9 ± 8	75.9 ± 14.2
Total	400	356	313	423	592	614	673	658
SFA	140.2 ± 13.1	76.6 ± 13.9	122.2 ± 9.9	135.4 ± 19.9	344.7 ± 84.1	255 ± 14.2	242.6 ± 30.8	236.1 ± 21.9
MUFA	91.9 ± 10.4	86.6 ± 26.4	67.5 ± 12.2	119.3 ± 20.8	129.8 ± 10.2	177.9 ± 11.7	202 ± 37.3	212.7 ± 16.4
PUFA	167.9 ± 26.2	192.8 ± 52.4	123.7 ± 22.9	167.7 ± 25.7	117.6 ± 26.4	181.1 ± 21.1	228.1 ± 23.7	209.7 ± 47.7
w-3	138.6 ± 22.2	159.7 ± 43.2	119.1 ± 22.4	105.2 ± 16.2	71.3 ± 9.6	74.6 ± 6.0	99.2 ± 13.2	103.1 ± 19.6
w-6	29.3 ± 4.0	33.1 ± 9.3	4.6 ± 0.5	62.5 ± 9.7	46.2 ± 16.8	98.5 ± 14.3	119.5 ± 11.1	98.8 ± 25.9

n.d.: not detected.

Table S5: Fatty acid composition of total lipids in the biomass of *Thraustochytrium* sp. RT2316-16 at various times during incubation. The biomass was grown at 15 °C in medium ML (glucose 5 g L^{-1}, yeast extract 12 g L^{-1}, monosodium glutamate 1.2 g L^{-1}).

Fatty acids	12 h	24 h	48 h	72 h	96 h	120 h	144 h	168 h
	(mg (g TL)$^{-1}$)							
C10:0	n.d.	n.d.	n.d.	n.d.	n.d.	n.d.	n.d.	n.d.
C12:0	n.d.	n.d.	7.4 ± 1.5	n.d.	n.d.	n.d.	n.d.	n.d.
C14:0	n.d.	n.d.	18.3 ± 6.2	9.0 ± 5.4	5.4 ± 1.9	n.d.	n.d.	n.d.
C14:1	n.d.	n.d.	n.d.	n.d.	n.d.	n.d.	n.d.	n.d.
C16:0	115.7 ± 36.5	51.0 ± 0.4	38.9 ± 16.6	47.5 ± 19.6	40.0 ± 1.3	31.0 ± 1.4	47.0 ± 7.2	46.5 ± 0.8
C16:1	n.d.	n.d.	13.5 ± 5.8	n.d.	7.9 ± 1.6	5.1 ± 0.1	n.d.	n.d.
C17:1	n.d.	n.d.	n.d.	n.d.	n.d.	n.d.	n.d.	n.d.
C18:0	23.3 ± 7.2	n.d.	43.9 ± 2.0	24 ± 1.2	17.3 ± 5.6	10.4 ± 0.2	22.5 ± 1.7	23.6 ± 1.8
C18:1n-9s	40.3 ± 9.6	25.5 ± 7.3	59.1 ± 2.7	52.8 ± 2.4	43.3 ± 2.9	24.4 ± 1.6	35.3 ± 12.9	28.9 ± 3.6
C18:2n-6	26.0 ± 7.1	11.4 ± 2.6	n.d.	12.1 ± 2.4	13.9 ± 2.6	7.7 ± 0.9	11.4 ± 4.5	7.5 ± 0.2
C18:3n-6	n.d.	n.d.	n.d.	n.d.	n.d.	n.d.	n.d.	n.d.
C20:1	n.d.	n.d.	n.d.	n.d.	n.d.	n.d.	n.d.	n.d.
C18:3n-3	n.d.	n.d.	n.d.	n.d.	n.d.	n.d.	n.d.	n.d.
C20:2	n.d.	n.d.	n.d.	n.d.	n.d.	n.d.	n.d.	n.d.
C20:3n-6	n.d.	n.d.	n.d.	n.d.	n.d.	n.d.	n.d.	n.d.
C22:1	n.d.	n.d.	9.0 ± 4.2	n.d.	n.d.	n.d.	n.d.	n.d.
C20:4n-6	n.d.	n.d.	n.d.	22.1 ± 3.9	n.d.	22 ± 1.4	26.7 ± 5.7	n.d.
C23:0	n.d.	11.2 ± 0.1	n.d.	n.d.	n.d.	n.d.	n.d.	n.d.
C24:0	n.d.	n.d.	n.d.	n.d.	n.d.	n.d.	n.d.	n.d.

C20:5n-3	75.9 ± 2.4	33.6 ± 0.1	17.8 ± 9.2	29 ± 5.2	44.3 ± 6.2	44.5 ± 0.6	56.3 ± 0.2	54.3 ± 11.7
C24:1	23.7 ± 7.4	24.2 ± 0.6	n.d.	18.4 ± 1.6	27.6 ± 0.2	27.2 ± 3.7	35.8 ± 7.7	37.2 ± 1.6
C22:5n-3	n.d.	n.d.	n.d.	9 ± 4.5	n.d.	n.d.	n.d.	n.d.
C22:6n-3	213.8 ± 30	140.4 ± 5.6	47.4 ± 21.6	93.6 ± 21.6	129 ± 14.8	132.3 ± 5.3	175.7 ± 4.5	184.1 ± 27.8
Total	519	297	255	318	329	305	411	382
SFA	139 ± 43.7	62.2 ± 0.5	108.5 ± 44.2	80.5 ± 37	62.6 ± 8.8	41.4 ± 1.7	69.5 ± 8.9	70.1 ± 2.7
MUFA	64 ± 16.9	49.8 ± 7.9	81.6 ± 33.7	71.3 ± 26	78.8 ± 4.7	56.7 ± 5.4	71.2 ± 20.6	66.1 ± 5.2
PUFA	315.7 ± 61.1	185.5 ± 8.3	65.2 ± 30.8	165.8 ± 37.6	187.2 ± 23.7	206.5 ± 8.3	270.1 ± 14.9	245.9 ± 39.7
w-3	289.7 ± 34.0	174.1 ± 5.6	65.2 ± 30.8	122.6 ± 26.7	173.3 ± 21.1	176.8 ± 6.0	232 ± 4.7	238.4 ± 39.4
w-6	26 ± 7.1	11.4 ± 2.6	n.d.	43.2 ± 10.9	13.9 ± 2.6	29.7 ± 2.3	38.1 ± 10.2	7.5 ± 0.2

n.d.: not detected.

Table S6: Fatty acid composition of total lipids in the biomass of *Thraustochytrium* sp. RT2316-16 at various times during incubation. The biomass was grown at 15 °C in medium MH (glucose 20 g L^{-1}, yeast extract 12 g L^{-1}, monosodium glutamate 1.2 g L^{-1}).

Fatty acids	12 h	24 h	48 h	72 h	96 h	120 h	144 h	168 h
	(mg (g TL)$^{-1}$)							
C10:0	n.d.	n.d.	2.8 ± 0.9	2.7 ± 1.1	3.7 ± 0.9	2.4 ± 0.2	1.8 ± 0.8	n.d.
C12:0	3.1 ± 0.9	n.d.	4.7 ± 1.8	6.9 ± 1.7	11.2 ± 1.4	7.5 ± 0.1	5.7 ± 2.2	5.9 ± 0.2
C14:0	15.0 ± 5.3	5.2 ± 0.9	11.0 ± 3.1	22.5 ± 5.9	28.8 ± 0.2	24.8 ± 2.8	17.3 ± 5.5	22.7 ± 4.8
C14:1	n.d.	n.d.	1.8 ± 0.5	3.8 ± 1	6.7 ± 0.5	5.3 ± 0.4	4.1 ± 1.9	5.2 ± 1.2
C16:0	92.7 ± 41.3	49.5 ± 18.1	24.3 ± 7.8	69.2 ± 20.1	73.7 ± 11.2	72.4 ± 14.3	51.6 ± 6.9	87.8 ± 34.6
C16:1	5.1 ± 7.2	n.d.	n.d.	n.d.	1.1 ± 1.6	1.5 ± 2.1	1.1 ± 1.6	2.0 ± 2.8
C17:1	n.d.	n.d.	n.d.	n.d.	n.d.	n.d.	n.d.	n.d.
C18:0	19.3 ± 11.2	5.8 ± 2.8	27.7 ± 6.5	83.3 ± 18.4	102.0 ± 1.2	54.1 ± 14	44.6 ± 24.7	66.0 ± 20.0
C18:1n-9	54.2 ± 26.7	32 ± 15.7	37.8 ± 5.3	88.7 ± 23.2	104.8 ± 21.9	100.6 ± 21.3	86.4 ± 13.7	148.5 ± 20.0
C18:2n-6	18.6 ± 11.4	7.6 ± 3.8	2.8 ± 0.3	9.8 ± 0.5	5.9 ± 1.5	24.6 ± 9.5	11.3 ± 2.5	20.1 ± 6.6
C18:3n-6	n.d.	0.5 ± 0.7	n.d.	n.d.	n.d.	n.d.	n.d.	n.d.
C20:1	n.d.	n.d.	0.8 ± 0	2.1 ± 0.7	2.8 ± 0.5	2.1 ± 0.3	1.9 ± 0.6	4.1 ± 1.7
C18:3n-3	n.d.	n.d.	n.d.	n.d.	n.d.	n.d.	n.d.	n.d.
C20:2	0.9 ± 1.2	n.d.	n.d.	1.8 ± 0	1.3 ± 0.4	3.1 ± 1.3	1.7 ± 0.1	4.4 ± 2.5
C20:3n-6	n.d.	n.d.	n.d.	n.d.	n.d.	n.d.	n.d.	n.d.
C22:1	0.6 ± 0.9	1.9 ± 1.2	3.2 ± 0.5	5 ± 0.2	4.5 ± 0.8	2.2 ± 0.4	2.3 ± 1.9	1.3 ± 0.3
C20:4n-6	n.d.	n.d.	n.d.	n.d.	n.d.	n.d.	n.d.	n.d.
C23:0	2.7 ± 2.9	6.6 ± 3.1	4.5 ± 0.8	12.9 ± 0.2	12.4 ± 1.6	20.3 ± 5.5	12.2 ± 2.0	25.7 ± 10.9
C24:0	1.0 ± 0.8	0.3 ± 0.4	0.7 ± 0.2	2.3 ± 0.8	3.1 ± 0.5	2.4 ± 0.8	1.7 ± 0.3	1.2 ± 1.7

C20:5n-3	4.7 ± 0.5	10 ± 4.4	11.9 ± 2.2	12.1 ± 1.2	10.7 ± 1.1	10.8 ± 2.4	8.2 ± 1.5	22.8 ± 8.2
C24:1	n.d.	n.d.	n.d.	n.d.	n.d.	n.d.	n.d.	n.d.
C22:5n-3	n.d.	n.d.	n.d.	n.d.	n.d.	n.d.	n.d.	n.d.
C22:6n-3	9.7 ± 11.5	30.2 ± 16.7	21.3 ± 3.9	31.8 ± 1.9	29.1 ± 2.8	31.7 ± 7.8	21.9 ± 3.9	65.0 ± 22.7
Total	228	150	155	355	402	366	274	483
SFA	133.9 ± 62.3	67.5 ± 25.3	75.8 ± 20.9	199.9 ± 48.2	234.9 ± 16.9	183.8 ± 37.7	134.9 ± 42.3	209.4 ± 72.2
MUFA	59.9 ± 34.8	33.9 ± 16.9	42.8 ± 6.3	97.5 ± 24.4	117.1 ± 24.9	109.7 ± 24.4	95.8 ± 19.7	161.0 ± 26.0
PUFA	33.8 ± 29.1	48.3 ± 25.7	36.0 ± 6.4	55.5 ± 3.6	47 ± 5.8	70.3 ± 21.0	43.1 ± 8.0	112.3 ± 40.0
w-3	14.4 ± 16.5	40.2 ± 21.2	33.2 ± 6.1	44 ± 3.1	39.8 ± 3.9	42.6 ± 10.2	30.1 ± 5.4	87.8 ± 30.9
w-6	18.6 ± 11.4	8.1 ± 4.5	2.8 ± 0.3	9.8 ± 0.5	5.9 ± 1.5	24.6 ± 9.5	11.3 ± 2.5	20.1 ± 6.6

n.d.: not detected.

Table S7: Fatty acid composition of total lipids in the biomass of *Thraustochytrium* sp. RT2316-16 at various times during incubation. The biomass was grown at 15 °C in medium M with glycerol as the carbon source.

Fatty acids	12 h	24 h	36 h	48 h	60 h	72 h	84 h	96 h
	(mg (g TL)$^{-1}$)							
C10:0	n.d.	n.d.	n.d.	n.d.	n.d.	0.8 ± 1.1	0.8 ± 1.1	n.d.
C12:0	n.d.	n.d.	n.d.	1.8 ± 2.6	3.1 ± 0.4	4 ± 0.3	4 ± 0.3	5.9 ± 1.0
C14:0	24.8 ± 0.1	6.5 ± 0.2	22.1 ± 2.1	24.3 ± 5.6	15.9 ± 2.4	32.8 ± 0.1	32.8 ± 0.1	21.4 ± 3.7
C14:1	n.d.	n.d.	n.d.	n.d.	n.d.	n.d.	n.d.	2.5 ± 0.5
C16:0	107.1 ± 0.2	49.5 ± 0.1	100.1 ± 13.9	77.1 ± 25.3	72.4 ± 9.2	165 ± 7.9	165 ± 7.9	74.7 ± 14.8
C16:1	n.d.	n.d.	5.7 ± 0.6	8.8 ± 2.7	2.7 ± 0.4	4.0 ± 0.2	4.0 ± 0.2	12.5 ± 2.4
C17:1	n.d.	n.d.	n.d.	n.d.	n.d.	n.d.	n.d.	1.1 ± 1.6
C18:0	35.4 ± 0.1	16.4 ± 0.1	93.9 ± 16.1	98.5 ± 31.4	73.7 ± 6.0	147.3 ± 9.1	147.3 ± 9.1	83 ± 21.8
C18:1n-9	55.5 ± 0.1	24.8 ± 0.2	90.4 ± 14	74.3 ± 27.6	46.8 ± 4.0	105.6 ± 6.4	105.6 ± 6.4	139.7 ± 33.4
C18:2n-6	18.7 ± 0.2	4.6 ± 0.1	n.d.	n.d.	6.8 ± 0.6	23.9 ± 1.3	23.9 ± 1.3	76.3 ± 1.8
C20:0	n.d.	n.d.	n.d.	n.d.	n.d.	1.3 ± 0.1	1.3 ± 0.1	n.d.
C18:3n-6	n.d.	n.d.	n.d.	n.d.	n.d.	n.d.	n.d.	n.d.
C20:2	n.d.	n.d.	n.d.	n.d.	n.d.	1.3 ± 0.1	1.3 ± 0.1	6.7 ± 2.0
C20:3n-6	n.d.	n.d.	n.d.	n.d.	n.d.	2.7 ± 0.2	2.7 ± 0.2	3.4 ± 1.1
C22:1	n.d.	n.d.	n.d.	n.d.	n.d.	2.2 ± 0.5	2.2 ± 0.5	12.1 ± 4.1
C20:4n-6	n.d.	n.d.	n.d.	n.d.	3.8 ± 0.1	2.2 ± 0.4	2.2 ± 0.4	4.1 ± 1.1
C24:0	n.d.	n.d.	n.d.	n.d.	n.d.	2 ± 0.2	2 ± 0.2	1.2 ± 1.6
C20:5n-3	n.d.	n.d.	n.d.	n.d.	7.4 ± 0.2	3.8 ± 0.7	3.8 ± 0.7	7.6 ± 1.5
C24:1	n.d.	n.d.	n.d.	n.d.	5.2 ± 0.1	4.8 ± 0.6	4.8 ± 0.6	4.7 ± 1.4

C22:5n-3	n.d.	n.d.	n.d.	n.d.	n.d.	n.d.	n.d.	n.d.
C22:6n-3	n.d.	n.d.	n.d.	n.d.	24.3 ± 0.5	12.7 ± 2.7	12.7 ± 2.7	29.1 ± 7.1
Total	242	102	312	285	262	516	516	486
SFA	167.4 ± 0.1	72.4 ± 0.1	216.1 ± 32.1	201.7 ± 64.9	165.1 ± 18.0	353 ± 18.7	353 ± 18.7	186.2 ± 42.9
MUFA	55.5 ± 0.3	24.8 ± 0.1	96.1 ± 14.6	83.1 ± 30.2	54.7 ± 4.6	116.6 ± 7.7	116.6 ± 7.7	172.5 ± 43.3
PUFA	18.7 ± 0.3	4.6 ± 0.5	n.d.	n.d.	42.4 ± 1.5	46.6 ± 5.4	46.6 ± 5.4	127.1 ± 30.7
w-3	n.d.	n.d.	n.d.	n.d.	24.3 ± 0.5	14.7 ± 2.9	14.7 ± 2.9	30.2 ± 8.7
w-6	18.7 ± 0.5	4.6 ± 0.5	n.d.	n.d.	10.7 ± 0.7	28.8 ± 1.9	28.8 ± 1.9	83.8 ± 20.1

n.d.: not detected.

Table S8: Fatty acid composition of total lipids in the biomass of *Thraustochytrium* sp. RT2316-16 at various times during incubation. The biomass was grown at 15 °C in medium M with canola oil as the carbon source.

Fatty acids	12 h	24 h	48 h	72 h	96 h	120 h	144 h	168 h	Canola oil
	(mg (g TL)$^{-1}$)								
C10:0	n.d.	n.d.	n.d.	n.d.	n.d.	n.d.	n.d.	n.d.	n.d.
C12:0	n.d.	n.d.	n.d.	n.d.	n.d.	n.d.	n.d.	n.d.	n.d.
C14:0	9.9 ± 3.3	n.d.	n.d.	n.d.	n.d.	n.d.	2.4 ± 0.9	n.d.	n.d.
C14:1	n.d.	n.d.	n.d.	n.d.	n.d.	n.d.	n.d.	n.d.	n.d.
C16:0	54.5 ± 4.7	39.9 ± 0.7	25.1 ± 3.6	17.2 ± 8.2	14.5 ± 3.2	32.4 ± 3.4	50.7 ± 7.7	49.4 ± 2.3	36.8 ± 3.0
C16:1	4.3 ± 0.7	n.d.	n.d.	n.d.	n.d.	2.8 ± 0.7	n.d.	3.8 ± 0.1	1.7 ± 0.2
C17:1	n.d.	n.d.	n.d.	n.d.	n.d.	n.d.	n.d.	n.d.	n.d.
C18:0	22.9 ± 1.3	11.9 ± 0.7	7.2 ± 1.1	3.3 ± 0.7	n.d.	5.3 ± 1.6	12.4 ± 2.9	12.4 ± 2.1	14.3 ± 1.2
C18:1n-9s	176.3 ± 50.0	247.1 ± 35.1	201 ± 19.1	105.3 ± 73.2	100.5 ± 37.3	249.2 ± 15.1	354.5 ± 34.1	310.2 ± 11.1	400.1 ± 32.8
C18:2n-6	71.9 ± 18.0	95.7 ± 14.6	80.2 ± 8.3	72.7 ± 50.4	58.5 ± 23.2	112 ± 11.4	151.2 ± 16	131.6 ± 0.9	169.4 ± 13.9
C18:3n-6	26.2 ± 9.7	41.6 ± 6.9	37 ± 3.9	43.5 ± 29.6	27.3 ± 11.1	8.6 ± 0.3	13.7 ± 0.7	11.9 ± 0.3	2.3 ± 0.2
C20:1	n.d.	5.3 ± 1.0	4.1 ± 0.6	n.d.	n.d.	6.4 ± 0.6	9.7 ± 1.1	8.4 ± 0.1	9.9 ± 0.9
C18:3n-3	n.d.	n.d.	n.d.	n.d.	n.d.	46.2 ± 4.3	61.8 ± 5.7	54.7 ± 0.6	77.8 ± 6.3
C20:2	n.d.	n.d.	n.d.	n.d.	n.d.	n.d.	2.2 ± 0.1	n.d.	n.d.
C20:3n-6	n.d.	n.d.	n.d.	n.d.	n.d.	n.d.	n.d.	n.d.	n.d.
C22:1	n.d.	n.d.	n.d.	n.d.	n.d.	n.d.	n.d.	n.d.	n.d.
C20:4n-6	n.d.	4.8 ± 0.7	n.d.	5.6 ± 2.8	3.5 ± 1.3	3.8 ± 1.2	3 ± 0.2	n.d.	n.d.
C23:0	n.d.	n.d.	n.d.	n.d.	n.d.	n.d.	n.d.	n.d.	n.d.
C24:0	n.d.	n.d.	n.d.	n.d.	n.d.	n.d.	1.8 ± 0.5	n.d.	n.d.

C20:5n-3	22 ± 2.8	19.2 ± 2.8	27 ± 9.9	34.8 ± 16.3	16.2 ± 9.3	14.1 ± 4.3	9.4 ± 1.6	10.3 ± 2	n.d.
C24:1	n.d.	5.4 ± 0.8	n.d.	n.d.	n.d.	5 ± 1.9	2.7 ± 0.3	n.d.	n.d.
C22:5n-3	n.d.	n.d.	n.d.	n.d.	n.d.	n.d.	4.4 ± 1.1	n.d.	n.d.
C22:6n-3	44.6 ± 5.7	37.8 ± 5.5	52.6 ± 21.8	79.2 ± 20	57.9 ± 20	57.3 ± 19.3	42.6 ± 5.1	41.6 ± 4.6	n.d.
Total	433	509	434	362	278	543	723	634	712
SFA	87.2 ± 9.3	51.8 ± 1.3	32.2 ± 4.7	20.5 ± 8.9	14.5 ± 3.2	37.7 ± 5.0	67.3 ± 12	61.8 ± 4.3	51.1 ± 4.3
MUFA	180.6 ± 50.6	252.5 ± 35.9	201 ± 19.1	105.3 ± 15.0	100.5 ± 37.3	257 ± 17.8	367 ± 35.6	322.4 ± 11.1	411.7 ± 33.9
PUFA	164.7 ± 36.2	199.1 ± 30.5	196.9 ± 43.9	235.8 ± 23.0	163.4 ± 25.7	195.8 ± 36.5	288.3 ± 30.5	250.2 ± 8.4	249.5 ± 20.4
w-3	66.6 ± 8.6	57.1 ± 8.4	79.7 ± 31.7	114 ± 36.3	74.1 ± 29.3	71.4 ± 23.6	52 ± 6.7	51.9 ± 6.6	n.d.
w-6	98.1 ± 27.7	142.1 ± 22.1	117.2 ± 12.2	121.8 ± 30	89.3 ± 35.6	124.4 ± 12.9	172.4 ± 18.1	143.6 ± 1.2	171.7 ± 14.1

n.d.: not detected.
